# Genomic diversity of culturable Paraburkholderia and Burkholderia species isolated from Bornean rainforest rhizosphere

**DOI:** 10.1099/mgen.0.001720

**Published:** 2026-05-15

**Authors:** Amal Alswat, Gordon Webster, Alex J. Mullins, Yoana D. Petrova, Benjamin J. Davies, Angharad R. Jones, James A.H. Murray, Andrew J. Weightman, Benoit Goossens, Eshwar Mahenthiralingam

**Affiliations:** 1 School of Biosciences, Cardiff University, Cardiff, Wales, CF10 3AX, UK; 2 Danau Girang Field Centre, c/o Sabah Wildlife Department, Wisma MUIS, Kota Kinabalu, Sabah, Malaysia

**Keywords:** Bornean rainforest, *Burkholderia*, genomic taxonomy, *Paraburkholderia*, rhizosphere

## Abstract

The genus *Paraburkholderia* was formally separated from *Burkholderia* in 2014 and now encompasses an expanding diversity of environmental species. To further resolve the taxonomy and functional potential of both genera, we conducted a comprehensive survey of 98 rhizosphere samples from the Bornean rainforest in Sabah, Malaysia, recovering 95 isolates spanning *Burkholderia* (*n*=15), *Paraburkholderia* (*n*=46) and other bacterial genera (*n*=34). Given the complexity of *Burkholderiaceae* classification, genome sequencing of 57 representative isolates coupled with average nucleotide identity (ANI) enabled accurate species assignment. ANI revealed that *Burkholderia* isolates comprised *Burkholderia cepacia* (*n*=7), *Burkholderia vietnamiensis* (*n*=2) and *Burkholderia diffusa*-like (*n*=3) taxa. Among *Paraburkholderia*, 22 isolates were assigned to *Paraburkholderia tropica*, eight to *Paraburkholderia bannensis* and single isolates to *Paraburkholderia heleia* and *Paraburkholderia guartelaensis*. Notably, 12 *Paraburkholderia* isolates represented putative novel species forming eight distinct genomic taxa. Although *Paraburkholderia* genomes encoded a broader repertoire of biosynthetic gene cluster classes, detectable antimicrobial activity and known bioactive metabolites (cepacin and pyrrolnitrin) were restricted to *Burkholderia*. In contrast, *Paraburkholderia* exhibited strong plant growth–promotion potential, with genomic predictions validated by phosphate solubilization, auxin production and extensive root-associated interactions. These findings reveal the Bornean rainforest rhizosphere as a rich reservoir of *Burkholderiaceae* diversity, particularly unexplored *Paraburkholderia* lineages.

**Impact Statement**

Our study provides a unique systematic genomic and phenotypic survey of culturable *Burkholderiaceae* from the Bornean rainforest rhizosphere. The survey revealed that a striking diversity of *Paraburkholderia* species was present including eight potentially novel taxa. A total of 57 high-quality genomes were generated from the rainforest *Burkholderiaceae*, significantly expanding the genomic resources available for environmental isolates of this diverse bacterial family, which has previously largely been studied for roles in infection. This demonstrated that rainforest *Paraburkholderia* encoded a broader repertoire of biosynthetic gene clusters than the recovered *Burkholderia* species, yet *in vitro* antimicrobial activity and the presence of known bioactive metabolites was restricted to the *Burkholderia*. The rainforest *Paraburkholderia* encoded multiple genomic plant growth-promoting traits including phosphate solubilization, auxin production and nitrogen fixation genes and also showed a highly active plant root colonization phenotype, resulting in endophytic interactions. Overall, this study demonstrates the importance of the tropical rainforest rhizosphere as a reservoir for *Burkholderiaceae* bacteria which show promise as agents for natural product discovery and crop enhancement.

## Data Summary

Additional information is provided in the manuscript text and supplementary data files. The bacterial genomes reported in this study have been submitted to the European Nucleotide Archive (ENA) under BioProject number PRJEB42734, and the accession numbers for the 57 genome sequenced strains are provided in Table 2 and are as follows: GCA-905219865, GCA-905219875, GCA-905219885, GCA-905220155, GCA-905219915, GCA-905220195, GCA-905220165, GCA-905220205, GCA-905220095, GCA-905220185, GCA-905220215, GCA-905220085, GCA-905220105, GCA-905219905, GCA-905219735, GCA-905219755, GCA-905219895, GCA-905219945, GCA-905219925, GCA-905219935, GCA-905219955, GCA-905219965, GCA-905219985, GCA-905220005, GCA-905220145, GCA-905220235, GCA-905220225, GCA-905219975, GCA-905219995, GCA-905219745, GCA-905219765, GCA-905219825, GCA-905219775, GCA-905220025, GCA-905220055, GCA-905220035, GCA-905220045, GCA-905220015, GCA-905220245, GCA-905220265, GCA-905220255, GCA-905220275, GCA-905219785, GCA-905220135, GCA-905220125, GCA-905220115, GCA-905220175, GCA-905220295, GCA-905219805, GCA-905219795, GCA-905219815, GCA-905220075, GCA-905219835, GCA-905219845, GCA-905220065, GCA-905220285 and GCA-905220305.

## Introduction

The Gram-negative bacterial genus *Burkholderia* was first proposed in 1992 as a novel taxonomic group that separated multiple species previously classified within the genera *Pseudomonas* [1]. *Burkholderia* encompassed bacterial strains and species that could be cultured from environmental and clinical sources and included primary human pathogens such as *Burkholderia pseudomallei* and the *Burkholderia cepacia* complex as opportunistic pathogens [2]. However, multiple *Burkholderia* were also found to be prolific in the rhizosphere of major global crop species and, up until the end of the 1990s, were harnessed as biopesticides or plant growth-promoting agents because of their agriculturally beneficial properties [3]. During the 2000s, there was greater recognition that certain species of *Burkholderia* were non-pathogenic, primarily associated with plants, and beneficial in the context of their ability to fix nitrogen and promote plant growth [4]. This led to the proposal by Sawana *et al.* [4] to split *Burkholderia* into two genera, with the new genus *Paraburkholderia*, encompassing generally environmental and plant beneficial species that were phylogenetically distinct from *Burkholderia sensu stricto* (which retained a mixture of pathogenic and environmental species). Using genomic taxonomy and Average Nucleotide Identity (ANI), a recent extensive study of 4,000 *Burkholderia sensu lato* genomes identified 137 named and 93 uncharacterized species within the multi-genus complex of *Burkholderia*, *Paraburkholderia*, *Trinickia*, *Caballeronia*, *Mycetohabitans*, *Robbsia* and *Pararobbisa* [5]. A key feature of *Burkholderia* and *Paraburkholderia* bacteria is their large genome size, on average 7.57 and 8.35 Mbp, respectively [5]. In addition, both genera have the capacity to encode biosynthetic gene clusters (BGCs) for a range of specialized metabolites [5], with the majority of characterized natural products attributed to *Burkholderia* [6].

Given the opportunistic pathogenicity shown by multiple *Burkholderia* species, there has been increased interest in characterizing *Paraburkholderia* for beneficial traits including bioremediation [7] and crop growth promotion [8]. Currently, there are 112 validly named *Paraburkholderia* species which have been isolated from a range of environmental sources [9]. They include well-characterized species such as *Paraburkholderia xenovorans* and its New York landfill isolated polychlorinated biphenyl-degrading strain, LB400, which has been extensively studied since its isolation in the 1980s [7]. *P. xenovorans* also represents one of the first *Burkholderiales* to be completely genome sequenced in 2006 [10]. *Paraburkholderia phytofirmans* is another well-studied species that was recovered from the onion rhizosphere and shows a wide range of plant-beneficial traits including the ability to promote plant growth via auxin production [11]. *Paraburkholderia phymatum* strain STM 815^T^ was isolated from root nodules of the legume *Mimosa pudica* and has also been extensively studied because it represented the first betaproteobacterium class to be capable of nodulation and nitrogen fixation (known as beta-rhizobia) [12]. A recent evolutionary study of *Paraburkholderia* genomes also estimated that they represent an ancient group of beta-rhizobia, with a most recent common ancestor emerging between 2,744 and 1,752 million years ago (Ma), *Paraburkholderia* itself evolving ~555 to 514 Ma, and subsequently acquiring its current nodulation loci between 103 and 48 Ma [13]. The study also suggested that rhizobial *Paraburkholderia* acquired their nodulation traits after the African and South American continents split and formed associations separately with the early lineages of leguminous plants that evolved ~110 to 65 Ma on these separate continents [13].

Although multiple species of *Paraburkholderia* have been characterized since its split from *Burkholderia* in 2014 [4], there have been few systematic surveys of environments from which *Paraburkholderia* can be isolated. In contrast, several studies have sought to characterize *B. cepacia* complex species within multiple environments such as the maize rhizosphere [14,15], industrial settings [16,17] and clinical infections in people with cystic fibrosis [18]. *Paraburkholderia* recovered from tropical forest environments include *P. phymatum* as the prototypic beta-rhizobia species recovered from *M. pudica* in the rainforest of French Guiana [12] and *Paraburkholderia nodosa* which was recovered from the Brazilian Atlantic Rainforest soil via legume trapping using the common bean, *Phaseolus vulgaris* L. [19]. In this study, we performed a unique survey of rhizosphere-associated *Paraburkholderia* and *Burkholderia* cultivated from rainforest on the island of Borneo, Sabah, Malaysia. A total 123 bacterial isolates were initially isolated with 57 undergoing whole-genome sequencing to accurately assign their taxonomy [5] and mine their genomes to understand specialized metabolite BGC diversity and encoded capacity for plant growth promotion (PGP). The survey enabled the limited recovery of *Burkholderia* species but resulted in the cultivation of multiple known and potentially novel *Paraburkholderia* species which phenotypically demonstrated PGP potential.

## Methods

### Sample site and plant rhizosphere collection for bacterial enrichment

Samples were collected in undisturbed rainforest floor areas around the forest trails at the Danau Girang Field Centre (DGFC; 05° 24.836′ N 118° 02.339′ E), in the Lower Kinabatangan Wildlife Sanctuary Sabah, Malaysia (Table 1, Fig. S1, available in the online Supplementary Material). DGFC is situated on the Kinabatangan River south of Sandakan, on the northeast coast of Borneo in the Malaysian State of Sabah and is surrounded by a mixture of lowland dipterocarp rainforest types. The field centre supports long-term conservation, facilities for higher education and scientific research, and is managed by Sabah Wildlife Department and Cardiff University, UK. Permission for sampling was obtained from the Sabah Wildlife Department.

**Table 1.** Sample site location and taxonomic classification of rhizosphere bacteria isolated from the rainforest at DGFC, Sabah, Malaysia

Rainforest isolate	Sampling site	Sampling location (latitude, longitude)	Identification by 16S rRNA gene similarity^†^	Identification by *recA* gene similarity^†^	Identification by genome ANI
J1-1	1	05° 24.836′ N 118° 02.339′ E	*Paraburkholderia* sp.	*Paraburkholderia tropica*	*Paraburkholderia tropica*
J1-2	1	05° 24.836′ N 118° 02.339′ E	*Paraburkholderia* sp.	*Paraburkholderia tropica*	*Paraburkholderia tropica*
J3	1	05° 24.836′ N 118° 02.339′ E	*Curtobacterium* sp.	–	–
J5	1	05° 24.836′ N 118° 02.339′ E	*Curtobacterium* sp.	–	–
J6	1	05° 24.836′ N 118° 02.339′ E	*Paraburkholderia* sp.	*Paraburkholderia tropica*	*Paraburkholderia tropica*
J7	1	05° 24.836′ N 118° 02.339′ E	*Paraburkholderia guartelaensis*	*Paraburkholderia* sp.	*Paraburkholderia* sp.
J8-1	1	05° 24.836′ N 118° 02.339′ E	*Paraburkholderia* sp.	*Paraburkholderia tropica*	*Paraburkholderia tropica*
J8-2	1	05° 24.836′ N 118° 02.339′ E	*Paraburkholderia acidiphila*	*Paraburkholderia* sp.	*Paraburkholderia* sp.
J8-8	1	05° 24.836′ N 118° 02.339′ E	*Enterococcus faecalis*	–	–
J10-1	1	05° 24.836′ N 118° 02.339′ E	*Paraburkholderia* sp.	*Paraburkholderia* sp.	*Paraburkholderia* sp.
J10-2	1	05° 24.836′ N 118° 02.339′ E	*Paraburkholderia guartelaensis*	*Paraburkholderia guartelaensis*	*Paraburkholderia guartelaensis*
J11-1	1	05° 24.836′ N 118° 02.339′ E	*Paraburkholderia* sp.	*Paraburkholderia* sp.	*Paraburkholderia bannensis*
J11-2	1	05° 24.836′ N 118° 02.339′ E	*Paraburkholderia acidiphila*	*Paraburkholderia* sp.	*Paraburkholderia* sp.
J12*	1	N05° 24.836′ N 118° 02.339′ E	*Paraburkholderia* sp.	*Paraburkholderia* sp.	*Paraburkholderia* sp.
J13	1	05° 24.836′ N 118° 02.339′ E	*Curtobacterium* sp.	–	–
J15-1	1	05° 24.836′ N 118° 02.339′ E	*Paraburkholderia* sp.	*Paraburkholderia* sp.	*Paraburkholderia bannensis*
J15-2	1	05° 24.836′ N 118° 02.339′ E	*Paraburkholderia* sp.	*Paraburkholderia* sp.	*Paraburkholderia bannensis*
J16	1	05° 24.836′ N 118° 02.339′ E	*Paraburkholderia* sp.	*Paraburkholderia tropica*	*Paraburkholderia tropica*
J17-1	2	05° 24.962′ N 118° 02.065′	*Burkholderia vietnamiensis*	*Burkholderia vietnamiensis*	*Burkholderia vietnamiensis*
J17-4	2	05° 24.820′ N 118° 02.332′ E	*Burkholderia vietnamiensis*	*Burkholderia vietnamiensis*	*Burkholderia vietnamiensis*
J18	2	05° 24.820′ N 118° 02.332′ E	*Curtobacterium* sp.	–	–
J19-1	2	05° 24.820′ N 118° 02.332′ E	*Paraburkholderia* sp.	*Paraburkholderia tropica*	*Paraburkholderia tropica*
J19-2	2	05° 24.820′ N 118° 02.332′ E	*Paraburkholderia* sp.	*Paraburkholderia tropica*	*Paraburkholderia tropica*
J20	2	05° 24.820′ N 118° 02.332′ E	*Klebsiella* sp.	–	–
J21	2	05° 24.820′ N 118° 02.332′ E	*Pantoea* sp.	–	–
J22	2	05° 24.820′ N 118° 02.332′ E	*Curtobacterium* sp.	–	–
J23-1	2	05° 24.820′ N 118° 02.332′ E	*Paraburkholderia* sp.	*Paraburkholderia tropica*	*Paraburkholderia tropica*
J23-2	2	05° 24.820′ N 118° 02.332′ E	*Paraburkholderia* sp.	*Paraburkholderia tropica*	*Paraburkholderia tropica*
J23-3	2	05° 24.820′ N 118° 02.332′ E	*Paraburkholderia* sp.	*Paraburkholderia tropica*	*Paraburkholderia tropica*
J24	2	05° 24.820′ N 118° 02.332′ E	*Paraburkholderia* sp.	*Paraburkholderia tropica*	*Paraburkholderia tropica*
J26	3	05° 24.725′ N 118° 02.384′ E	*Paraburkholderia* sp.	*Paraburkholderia tropica*	*Paraburkholderia tropica*
J27	3	05° 24.725′ N 118° 02.384′ E	*Paraburkholderia* sp.	*Paraburkholderia tropica*	*Paraburkholderia tropica*
J30	3	05° 24.725′ N 118° 02.384′ E	*Curtobacterium* sp.	–	–
J33	3	05° 24.725′ N 118° 02.384′ E	*Leifsonia* sp.	–	–
J34-3	3	05° 24.725′ N 118° 02.384′ E	*Klebsiella* sp.	–	–
J35	4	05° 24.619′ N 118° 02.443′ E	*Paraburkholderia bannensis*	*Paraburkholderia* sp.	*Paraburkholderia bannensis*
J36	4	05° 24.619′ N 118° 02.443′ E	*Curtobacterium* sp.	–	–
J36-1	4	05° 24.619′ N 118° 02.443′ E	*Leifsonia* sp.	–	–
J38^‡^	4	05° 24.619′ N 118° 02.443′ E	*Pandoraea* sp.	–	–
J41	4	05° 24.619′ N 118° 02.443′ E	*Paraburkholderia* sp.	*Paraburkholderia* sp.	*Paraburkholderia* sp.
J41-2	4	05° 24.619′ N 118° 02.443′ E	*Curtobacterium* sp.	–	–
J42	4	05° 24.619′ N 118° 02.443′ E	*Paraburkholderia heleia*	*Paraburkholderia heleia*	*Paraburkholderia heleia*
J45-1	5	05° 24.840′ N 118° 02.239′ E	*Paraburkholderia* sp.	*Paraburkholderia tropica*	*Paraburkholderia tropica*
J45-2	5	05° 24.840′ N 118° 02.239′ E	*Paraburkholderia* sp.	*Paraburkholderia tropica*	*Paraburkholderia tropica*
J47-2	5	05° 24.840′ N 118° 02.239′ E	*Burkholderia* sp.	*B. cepacia*	*B. cepacia*
J47-3	5	05° 24.840′ N 118° 02.239′ E	*Burkholderia* sp.	*B. cepacia*	*B. cepacia*
J48	5	05° 24.840′ N 118° 02.239′ E	*Burkholderia* sp.	*Burkholderia* sp.	*Burkholderia diffusa*
J49	5	05° 24.840′ N 118° 02.239′ E	*Burkholderia* sp.	*B. cepacia*	*B. cepacia*
J50-1	5	05° 24.840′ N 118° 02.239′ E	*Paraburkholderia* sp.	*Paraburkholderia tropica*	*Paraburkholderia tropica*
J50-2	5	05° 24.840′ N 118° 02.239′ E	*Paraburkholderia* sp.	*Paraburkholderia tropica*	*Paraburkholderia tropica*
J50-3	5	05° 24.840′ N 118° 02.239′ E	*Paraburkholderia* sp.	*Paraburkholderia tropica*	*Paraburkholderia tropica*
J50-4	5	05° 24.840′ N 118° 02.239′ E	*Paraburkholderia* sp.	*Paraburkholderia tropica*	*Paraburkholderia tropica*
J52	6	05° 24.871′ N 118° 02.139′ E	*Curtobacterium* sp.	–	–
J53	6	05° 24.871′ N 118° 02.139′ E	*Enterobacter* sp.	–	–
J54	6	05° 24.871′ N 118° 02.139′ E	*Enterobacter* sp.	–	–
J56	6	05° 24.871′ N 118° 02.139′ E	*Curtobacterium* sp.	–	–
J58	6	05° 24.871′ N 118° 02.139′ E	*Curtobacterium* sp.	–	–
J59	6	N05° 24.871′ N 118° 02.139′ E	*Klebsiella* sp.	–	–
J61	7	05° 24.850′ N 118° 02.395′ E	*Brucella* sp.	–	–
J62	7	05° 24.850′ N 118° 02.395′ E	*Paraburkholderia* sp.	*Paraburkholderia tropica*	*Paraburkholderia tropica*
J63	7	05° 24.850′ N 118° 02.395′ E	*Paraburkholderia* sp.	*Paraburkholderia* sp.	*Paraburkholderia* sp.
J64	7	05° 24.850′ N 118° 02.395′ E	*Bacillus* sp.	–	–
J65	7	05° 24.850′ N 118° 02.395′ E	*Enterobacter* sp.	–	–
J66	7	05° 24.850′ N 118° 02.395′ E	*Lactococcus* sp.	–	–
J67	7	05° 24.850′ N 118° 02.395′ E	*Paraburkholderia* sp.	*Paraburkholderia* sp.	*Paraburkholderia* sp.
J69-1	7	05° 24.850′ N 118° 02.395′ E	*Paraburkholderia* sp.	*Paraburkholderia* sp.	*Paraburkholderia* sp.
J69-2	7	05° 24.850′ N 118° 02.395′ E	*Paraburkholderia* sp.	*Paraburkholderia* sp.	*Paraburkholderia* sp.
J70	7	05° 24.850′ N 118° 02.395′ E	*Burkholderia* sp.	*B. cepacia*	*B. cepacia*
J72*	7	05° 24.850′ N 118° 02.395′ E	*Paraburkholderia bannensis*	*Paraburkholderia* sp.	*Paraburkholderia bannensis*
J73	7	05° 24.850′ N 118° 02.395′ E	*Curtobacterium* sp.	–	–
J74	8	N05° 24.850′ N E118° 02.299′ E	*Paraburkholderia* sp.	*Paraburkholderia* sp.	*Paraburkholderia bannensis*
J75-1	8	N05° 24.850′ N E118° 02.299′ E	*Paraburkholderia* sp.	*Paraburkholderia* sp.	*Paraburkholderia bannensis*
J75-2	8	N05° 24.850′ N E118° 02.299′ E	*Paraburkholderia bannensis*	*Paraburkholderia* sp.	*Paraburkholderia bannensis*
J76	8	N05° 24.850′ N E118° 02.299′ E	*Paraburkholderia* sp.	*Paraburkholderia* sp.	*Paraburkholderia* sp.
J76-1	8	N05° 24.850′ N E118° 02.299′ E	*Pantoea* sp.	–	–
J78-1^‡^	8	N05° 24.850′ N E118° 02.299′ E	*Paraburkholderia* sp.	*Paraburkholderia tropica*	–
J78-2	8	N05° 24.850′ N E118° 02.299′ E	*Curtobacterium* sp.	–	–
J78-4	8	N05° 24.850′ N E118° 02.299′ E	*Pantoea* sp.	–	–
J78-5^‡^	8	N05° 24.850′ N E118° 02.299′ E	*Burkholderia* sp.	*Burkholderia* sp.	–
J78-9^‡^	8	N05° 24.850′ N E118° 02.299′ E	*Burkholderia* sp.	*Burkholderia* sp.	–
J78-10^‡^	8	N05° 24.850′ N E118° 02.299′ E	*Burkholderia* sp.	*Burkholderia* sp.	–
J80-2	8	N05° 24.850′ N E118° 02.299′ E	*Burkholderia* sp.	*B. cepacia*	*B. cepacia*
J85	8	N05° 24.850′ N E118° 02.299′ E	*Klebsiella* sp.	–	–
J86-1	8	N05° 24.850′ N E118° 02.299′ E	*Burkholderia* sp.	*B. cepacia*	*B. cepacia*
J86-2	8	N05° 24.850′ N E118° 02.299′ E	*Burkholderia* sp.	*B. cepacia*	*B. cepacia*
J87	8	N05° 24.850′ N E118° 02.299′ E	*Klebsiella* sp.	–	–
J88	8	N05° 24.850′ N E118° 02.299′ E	*Paraburkholderia* sp.	*Paraburkholderia tropica*	*Paraburkholderia tropica*
J89	9	05° 24.609′ N and 118° 02.411′ E	*Lactococcus* sp.	–	–
J91-1	9	05° 24.609′ N and 118° 02.411′ E	*Burkholderia* sp.	*Burkholderia* sp*.*	*Burkholderia diffusa*
J91-2	9	05° 24.609′ N and 118° 02.411′ E	*Burkholderia* sp.	*Burkholderia* sp*.*	*Burkholderia diffusa*
J92	9	05° 24.609′ N and 118° 02.411′ E	*Paraburkholderia* sp.	*Paraburkholderia tropica*	*Paraburkholderia tropica*
J94*	9	05° 24.609′ N and 118° 02.411′ E	*Paraburkholderia* sp.	*Paraburkholderia* sp.	*Paraburkholderia* sp.
J95	9	05° 24.609′ N and 118° 02.411′ E	*Pantoea* sp.	–	–
J97	9	05° 24.609′ N and 118° 02.411′ E	*Caballeronia zhejiangensis*	*Caballeronia* sp.	*Caballeronia* sp.
J98^‡^	9	05° 24.609′ N and 118° 02.411′ E	*Paraburkholderia* sp.	*Paraburkholderia* sp.	–

*Rainforest isolates were isolated from plant rhizosphere soil with the exception of J12 and J72 which were isolated from a soil covered with fungi, and J94 was isolated from soil underneath dead wood.

†Rainforest isolates were putatively identified by 16S rRNA and/or *recA* gene sequencing using the following criteria: >99% sequence similarity for species, >95% sequence similarity for genus with >95% sequence query coverage. Fifty-seven *Burkholderiacea* isolates were then further identified by genome sequencing (see Table 2) and confirmed by ANI.

‡Isolates J38, J78-1, J78-5, J78-9, J78-10 and J98 did not revive after storage at −80 °C.

**Table 2.** Genome assembly summary metrics of the 57 rainforest rhizosphere *Burkholderiacea* isolates

Rainforest isolate	Genome accession no.	BCC strain no.	Genomic species assignment	Genome size (Mbp)	Sequencing depth (x coverage)	No. of contigs >1,000 bp (total no.)	N_50_ (bp)	G+C content (%)	Genome completeness (%)	No. of CDS
***Burkholderia* species isolates (*n*=12**)										
J47-2	GCA-905219745	BCC1959	*B. cepacia*	8.3	90	76 (156)	201,276	66.9	99.1	7,658
J47-3	GCA-905219765	BCC1958	*B. cepacia*	8.5	119	74 (136)	222,912	66.8	99.3	7,890
J49	GCA-905219775	BCC1967	*B. cepacia*	8.5	130	68 (126)	258,240	66.8	99.3	7,884
J70	GCA-905219785	BCC1960	*B. cepacia*	8.7	126	147 (335)	181,965	66.5	99.1	8,177
J80-2	GCA-905219805	BCC1963	*B. cepacia*	8.9	99	82 (172)	239,734	66.5	99.1	8,237
J86-1	GCA-905219795	BCC1962	*B. cepacia*	8.8	93	88 (177)	229,690	66.5	99.4	8,206
J86-2	GCA-905219815	BCC1961	*B. cepacia*	8.8	80	92 (195)	212,588	66.5	99.4	8,215
J48	GCA-905219825	BCC1957	*Burkholderia diffusa*	7.3	142	59 (101)	223,626	66.5	99.5	6,750
J91-1	GCA-905219835	BCC1965	*Burkholderia diffusa*	7.2	129	74 (135)	217,336	66.5	99.5	6,604
J91-2	GCA-905219845	BCC1964	*Burkholderia diffusa*	7.2	138	75 (136)	217,336	66.5	99.5	6,605
J17-1	GCA-905219735	BCC1956	*Burkholderia vietnamiensis*	6.9	125	98 (179)	155,444	67.1	99.9	6,285
J17-4	GCA-905219755	BCC1955	*Burkholderia vietnamiensis*	6.9	108	72 (113)	200,345	67.1	99.9	6,293
**Mean±sd**	–	–	–	**8.00±0.81**	–	–	–	**66.7±0.24**	–	**7,400±815**
***Paraburkholderia* species isolates (*n*=44)**										
J11-1	GCA-905220095	BCC1941	*Paraburkholderia bannensis*	8.8	111	40 (80)	540,172	63.5	99.6	7,860
J15-1	GCA-905220085	BCC1939	*Paraburkholderia bannensis*	8.9	93	61 (105)	363,282	63.5	99.8	7,870
J15-2	GCA-905220105	BCC1938	*Paraburkholderia bannensis*	8.5	128	39 (84)	641,780	63.9	99.2	7,553
J35	GCA-905220145	BCC1932	*Paraburkholderia bannensis*	8.6	113	47 (113)	343,593	63.5	99.8	7,729
J72	GCA-905220135	BCC1917	*Paraburkholderia bannensis*	8.7	79	84 (180)	166,145	63.5	99.4	7,832
J74	GCA-905220125	BCC1916	*Paraburkholderia bannensis*	8.8	99	57 (112)	374,821	63.5	99.8	7,913
J75-1	GCA-905220115	BCC1915	*Paraburkholderia bannensis*	8.6	76	44 (76)	501,129	63.8	99.8	7,603
J75-2	GCA-905220175	BCC1914	*Paraburkholderia bannensis*	8.8	102	35 (72)	768,013	63.5	99.4	7,805
J10-2	GCA-905220205	BCC1942	*Paraburkholderia guartelaensis*	9.3	86	122 (190)	308,842	63.5	99.7	8,439
J42	GCA-905220225	BCC1930	*Paraburkholderia heleia*	7.8	111	89 (149)	255,736	64.5	99.2	7,250
J7	GCA-905220155	BCC1912	*Paraburkholderia* sp.	9	65	76 (192)	279,902	63.9	99.6	8,171
J8-2	GCA-905220195	BCC1943	*Paraburkholderia* sp.	9.5	66	101 (181)	272,312	63.5	99.6	8,669
J10-1	GCA-905220165	BCC1954	*Paraburkholderia* sp.	9.1	224	55 (112)	448,252	63.9	99.6	8,195
J11-2	GCA-905220185	BCC1940	*Paraburkholderia* sp.	9.2	88	89 (187)	236,207	63.8	99.6	8,342
J12	GCA-905220215	BCC1953	*Paraburkholderia* sp.	6.9	97	43 (66)	344,856	64.9	99.2	6,330
J41	GCA-905220235	BCC1931	*Paraburkholderia* sp.	7	140	88 (146)	187,711	65.1	99.7	6,352
J63	GCA-905220245	BCC1922	*Paraburkholderia* sp.	8.8	97	130 (284)	169,965	65.1	99.7	7,892
J67	GCA-905220265	BCC1921	*Paraburkholderia* sp.	9.4	100	81 (189)	274,084	63.5	99.6	8,335
J69-1	GCA-905220255	BCC1920	*Paraburkholderia* sp.	9	94	71 (128)	367,675	62.5	99.2	8,147
J69-2	GCA-905220275	BCC1919	*Paraburkholderia* sp.	9	68	75 (137)	253,607	62.5	99.2	8,147
J76	GCA-905220295	BCC1913	*Paraburkholderia* sp.	7.1	117	42 (76)	357,328	64.5	99.3	6,392
J94	GCA-905220285	BCC1909	*Paraburkholderia* sp.	8.5	83	45 (83)	447,235	65	99.7	7,554
J1-1	GCA-905219865	BCC1935	*Paraburkholderia tropica*	8.6	86	73 (127)	316,706	64.8	99.2	7,714
J1-2	GCA-905219875	BCC1936	*Paraburkholderia tropica*	8.6	84	63 (95)	316,900	64.8	99.2	7,720
J6	GCA-905219885	BCC1918	*Paraburkholderia tropica*	8	70	63 (112)	265,916	65.1	99.2	7,034
J8-1	GCA-905219915	BCC1949	*Paraburkholderia tropica*	8.3	91	70 (110)	258,927	64.9	99.2	7,425
J16	GCA-905219905	BCC1937	*Paraburkholderia tropica*	8	90	67 (120)	266,003	65.1	99.2	7,076
J19-1	GCA-905219895	BCC1950	*Paraburkholderia tropica*	8.5	112	76 (121)	310,799	64.9	99.2	7,583
J19-2	GCA-905219945	BCC1951	*Paraburkholderia tropica*	8.5	111	76 (118)	310,799	64.9	99.2	7,582
J23-1	GCA-905219925	BCC1948	*Paraburkholderia tropica*	8.5	112	99 (160)	258,721	64.9	99.2	7,638
J23-2	GCA-905219935	BCC1947	*Paraburkholderia tropica*	8.5	107	90 (150)	258,721	64.9	99.2	7,631
J23-3	GCA-905219955	BCC1946	*Paraburkholderia tropica*	8.5	133	94 (156)	258,721	64.9	99.2	7,631
J24	GCA-905219965	BCC1934	*Paraburkholderia tropica*	8.4	122	71 (98)	287,287	64.9	99.2	7,464
J26	GCA-905219985	BCC1933	*Paraburkholderia tropica*	8.4	105	71 (99)	334,594	64.9	99.2	7,461
J27	GCA-905220005	BCC1945	*Paraburkholderia tropica*	8.5	122	92 (154)	258,721	64.9	99.2	7,628
J45-1	GCA-905219975	BCC1929	*Paraburkholderia tropica*	7.9	124	53 (80)	347,688	65.1	99.2	6,988
J45-2	GCA-905219995	BCC1928	*Paraburkholderia tropica*	8.1	133	65 (147)	244,908	65	99.2	7,164
J50-1	GCA-905220025	BCC1927	*Paraburkholderia tropica*	8.3	104	97 (211)	223,578	64.9	99.2	7,411
J50-2	GCA-905220055	BCC1926	*Paraburkholderia tropica*	8.3	126	98 (220)	187,754	64.9	99.2	7,405
J50-3	GCA-905220035	BCC1925	*Paraburkholderia tropica*	8.3	112	86 (156)	244,042	64.9	99.2	7,390
J50-4	GCA-905220045	BCC1924	*Paraburkholderia tropica*	8.3	102	86 (141)	280,271	64.9	99.2	7,388
J62	GCA-905220015	BCC1923	*Paraburkholderia tropica*	8.2	94	87 (149)	200,970	64.9	99.2	7,400
J88	GCA-905220075	BCC1911	*Paraburkholderia tropica*	8.6	92	80 (150)	223,564	64.5	99.2	7,576
J92	GCA-905220065	BCC1910	*Paraburkholderia tropica*	8.3	97	65 (107)	309,465	64.9	99.2	7,478
**Mean±sd**	–	–	–	**8.48±0.55**	–	–	–	**64.38±0.74**	–	**7,595±503**
***Caballeronia* species isolate**										
J97	GCA-905220305	BCC1952	*Caballeronia* sp.	7.8	103	85 (135)	199,475	63.5	99.5	7,227

CDS, coding sequence.

A total of 98 plant rhizosphere samples were collected from nine locations and different plant species around the DGFC forest area (Fig. S1B) between 23 and 25 August 2008 (prior to the Nagoya Protocol). Rhizosphere samples (2–5 cm roots) were collected from small plants on the rainforest floor under aseptic conditions, using ethanol-washed stainless-steel spatulas and scissors, labelled by location and placed into grip-seal polythene plastic bags. Surrounding soil was shaken off the root systems as far as possible, but they were not washed to remove any adherent soil. All samples were processed by selecting a tap root or lateral root ~2.0 to 5.0 cm from the stem base of each plant and cutting this region into 1.0 cm length pieces. A single section of root was placed in sterile 1.5 ml microcentrifuge tubes (Fisher Scientific) containing 1.0 ml basal salt medium with glycerol (BSMG) [20] supplemented with 100 µg ml^−1^ cycloheximide. Each sample was homogenized with a sterile microtube pestle [Starlab (UK) Ltd] and incubated at ambient temperature (~30 °C) for 2 days at DGFC. All BSMG-enriched culture samples were then transported to Cardiff University, Wales, UK (~10 days) and cryopreserved by addition of 8% (v/v) DMSO and stored at −80 °C.

### Isolation of rainforest rhizosphere *Burkholderiaceae* bacteria using *Pseudomonas cepacia* azelaic acid tryptamine media

All BSMG enrichments were allowed to thaw for 30 min, and 100 µl of each enrichment culture was inoculated into 3.0 ml of the *Burkholderia* semi-selective *P. cepacia* azelaic acid tryptamine (PCAT; pH 5.7) media [21] contained in 15 ml polypropylene centrifuge tubes (Sarstedt AG and Co. KG) and incubated for 3 days at 30 °C on a rocking platform (50 r.p.m.). Enrichment cultures showing growth on PCAT (88 out of 98) were serially diluted, and the 10^−4^ to 10^−6^ dilutions were spread plated using an automated spiral plater (Don Whitley Scientific) onto 1.5% (w/v) agar-solidified PCAT media. Agar plates were incubated at 30 °C for a further 3 days. A total of 123 single colonies were selected based on the presence of different colony morphotypes that were observed across the samples being processed and were sub-cultured three times on tryptone soya agar (Oxoid Limited) for purity and stored in 8% (v/v) DMSO at −80 °C.

### 16S rRNA and *recA* gene phylogenetic analysis of rainforest rhizosphere *Burkholderiaceae* isolates

DNA was extracted from all 123 bacterial isolates. For each isolate, 5.0 ml of an overnight culture was grown in tryptone soya broth (TSB; Oxoid Limited) at 30 °C on a rocking platform (50 r.p.m.) for 12–16 h overnight. Bacterial cells were harvested by centrifugation at 4,000 r.p.m. using an ALC-PK120 Centrifuge for 10 min, and gDNA was extracted using a Maxwell® 16 Instrument and Tissue DNA purification Kit (Promega) according to the manufacturer’s instructions. Extracted gDNA was used as template in a 16S rRNA gene PCR with bacterial primers 27F/1492R [22] and a *recA* gene PCR with primers BUR1/BUR2 [23] using DreamTaq Green PCR master mix (Thermo Fisher Scientific). All 16S rRNA and *recA* gene PCR amplicons were analysed by 1.2% (w/v) agarose gel electrophoresis, purified with Monarch PCR and DNA Cleanup Kit (NEB) and sequenced at Eurofins Genomics (https://www.eurofinsgenomics.eu/en/home/) by Sanger sequencing with primers 27F or BUR1. Sequence chromatograms were analysed using Chromas v2.6.6 (http://technelysium.com.au) and identified by Nucleotide blast implemented on the NCBI server (https://blast.ncbi.nlm.nih.gov/Blast.cgi) against the nucleotide collection (core_nt) database, and non-*Burkholderiaceae* species were removed from the culture collection. DNA from isolates that were putatively identified as *Burkholderia* or *Paraburkholderia* species was amplified by random amplified polymorphic DNA (RAPD)-PCR using primer 270 5′-TGCGCGCGGG-3′ as described [24] to reduce the number of duplicated isolates within the collection (Fig. S2).

Bacterial 16S rRNA and *recA* gene sequences from 57 sequenced genomes were further analysed using Nucleotide blast against the (core_nt) and, for the 16S rRNA genes only, against the reference RNA sequences (refseq_rna) databases to identify closest relatives and reference sequences. All 16S rRNA and *recA* gene sequences were aligned using MAFFT v7 online [25] with sequences retrieved from the database. Alignments were edited manually using BioEdit [26], and phylogenetic trees were constructed using mega X [27] by using minimum evolution and LogDet methods with 1,000 bootstraps. Congruent trees were also obtained using other methods, including maximum likelihood method with the general time reversible model and gamma distribution and neighbour joining with the Jukes–Cantor algorithm.

### Bacterial genome sequencing and assembly

Bacterial gDNA was extracted from isolates of interest (*n*=57), previously identified by 16S rRNA gene sequencing, from a 3.0 ml overnight culture grown in TSB at 30 °C. Each genome-sequenced isolate was also assigned a ‘BCC’ number for archiving within the Cardiff University *Burkholderia* Culture Collection (Table 2). Cells were collected by centrifugation and DNA extracted as described above. DNA was quantified using a Qubit 3.0 Fluorometer and a Qubit dsDNA Broad Range (BR) Assay Kit (Thermo Fisher Scientific). Libraries were prepared for 250 bp nucleotide paired-end sequencing using the NEBNext® Ultra II DNA Library Prep Kit for Illumina. Genome libraries were then sequenced by an Illumina MiSeq platform. Sequence reads were trimmed from Illumina adaptors using the TrimGalore v0.4.2 script (https://www.bioinformatics.babraham.ac.uk/projects/trim_galore/) and paired reads were merged with FLASH v1.2.11 [28]. Genomes were assembled with SPAdes v3.13.0 [29], and mis-assemblies were corrected using the software Pilon v1.22 [30]. Genome annotation was done using the NCBI Prokaryotic Genome Annotation Pipeline [31] after submission to the public database. Bacterial genome sequences reported in this study have been submitted to the European Nucleotide Archive (ENA) under the project/study accession number PRJEB42734. All genome accession numbers are listed in Table 2.

### Species identification of *Burkholderiaceae* genomes

To allow species identification of the 57 bacterial genomes, the genus of each bacterial isolate was initially assigned by 16S rRNA gene sequencing coupled with genome identification using the taxonomic sequence classification system Kraken2 v2.0.6-beta [32] and RefSeq complete bacterial genomes [33]. Using this preliminary identification as a guide, full species assignments were subsequently achieved by comparing ANI [34] against reference genomes. Bacterial isolate and type strain genome sequences were passed to an alignment-based ANI tool, PyANI-plus for enhanced ANI accuracy and calculated using ANIm [35]. In addition, genome sequences were also uploaded to the Type (Strain) Genome Server (TYGS) (https://tygs.dsmz.de) for whole genome-based taxonomic analysis [36] and the Genome-to-Genome Distance Calculator (GGDC) (https://ggdc.dsmz.de/home.php) for digital DNA–DNA hybridization (dDDH) [37].

### Genomic assessment of BGCs and PGP potential

Specialized metabolite BGCs were predicted with antiSMASH v7.1.0 [38], and coding sequences (CDSs) were predicted using Prodigal v2.6.3 [39]. The presence of polyyne BGCs was determined by protein–protein blast v2.14.0+ of the cepacin desaturase protein gene (*ccnN*) [40] against the CDSs of the genome collection. Determination of potential PGP properties was carried out by searching the PGAP genome annotations [31] through the NCBI Datasets resource (https://www.ncbi.nlm.nih.gov/datasets/) with filters set for nitrogen fixation (*nif*, *fix* and *fdx* genes), phosphate solubilization (*pqq* genes) and auxin production (*iaaH*). Selected PGP genes were chosen based on previous studies on plant growth-promoting bacteria [41].

### *In vitro* microbial antagonism assays and HPLC detection of specialized metabolites

Antagonism assays for rainforest isolates were performed against a panel of susceptibility organisms: *Staphylococcus aureus* NCTC 12981 (Gram-positive bacterium), *Pectobacterium carotovorum* LMG 2464 (Gram-negative bacterium) and *Candida albicans* SC 5314 (fungus) as described [20,42]. Briefly, bacterial isolates were grown overnight at 30 °C in TSB, spotted (2.0 µl bacteria) onto BSMG (pH 5 or pH 7) agar plates and incubated at 22 °C for 3 days. The resulting microbial colony was killed by chloroform exposure, overlaid with susceptibility organism-seeded [0.4%(v/v)] half-strength iso-sensitest agar (Oxoid Limited), and the plate was incubated at 30 °C for 24 h. Plates were then examined for zones of inhibition [20].

Specialized metabolites were detected using the HPLC-based screening method described previously [43]. Rainforest isolates were streaked onto BSMG (pH 7) plates and incubated for 3 days at 22 °C. Following incubation, bacterial growth was removed, and a 20 mm disc was cut from the centre of the plate. The agar disc was placed into a 30 ml wide-mouth amber glass bottle with 0.5 ml dichloromethane (DCM) and agitated on a rocking platform (40 r.p.m.) for 2 h. DCM extracts were analysed by HPLC on a Waters® AutoPurification™ HPLC system fitted with a reversed-phase analytical column (Waters® XSelect CSH C18, 4.6×100 mm, 5 µm) and a C18 SecurityGuard™ cartridge (Phenomenex) in series. Detection of compounds was by absorbance at 210–400 nm by a photo-diode array detector [43].

### PGP and endophytic interaction assays

The production of indole-3-acetic acid (IAA) by a selection of *Paraburkholderia* was analysed by using an adapted Salkowski’s reagent protocol [44]. Essentially, bacteria were grown overnight in TSB, washed, resuspended in PBS and adjusted to OD_600_=1.0. For each isolate tested, 50 µl of PBS suspension was added to 6 ml of yeast malt medium (YM) [41] supplemented with 100 mg ml^−1^ of l-tryptophan and incubated at 30 °C for 4 days on a rocking platform (50 r.p.m.). After incubation, cultures were centrifuged at 4,000 r.p.m. (ALC-PK120 centrifuge) for 10 min, and 500 µl of cell-free supernatant was mixed with 1 ml of Salkowski’s reagent (50 ml: 35% (v/v) perchloric acid; 1 ml 0.5 M FeCL_3_) and 25 µl of 10 mM phosphoric acid. Samples were incubated in the dark at room temperature (RT) for 25 min. After incubation, 200 µl was added to a 96-well plate in triplicate, and absorbance was measured at 530 nm using a Tecan Infinite M200PRO. The concentration of IAA was determined using a standard curve of IAA (Fisher Scientific) ranging from 0 to 20 µg ml^−1^ in YM.

Inorganic phosphate solubilization was qualitatively assessed using National Botanical Research Institute’s phosphate solubilization agar (NBRIP) [45]. Bacteria were grown in TSB, washed and resuspended in PBS as above. Isolates (10 µl) were spotted on NBRIP agar plates and incubated at 30 °C for 11 days. After incubation, the diameter of the clear zone around the colony was measured (mm) to estimate inorganic phosphate solubilization. *In vitro* plant germination assays [*Medicago sativa* (alfalfa); Kings Seeds] were adapted from Altier *et al.* [46]. Alfalfa seeds were surface sterilized in 5% NaOCl (~45–50 ml for 300 seeds) in 50 ml Falcon tubes for 20 min and washed five times in sterile water (~50 ml each time). Sterilized seeds (24 per plate) were dipped in a PBS-bacterial suspension (prepared as above) and placed on 1% (w/v) water agar (purified agar; Oxoid Limited) in 9.0 cm Petri dishes. Inoculated seeds were incubated at 22 °C in the dark for 3 days, followed by incubation in the light at RT for a further 7 days. The effect of rainforest isolates on the growth of alfalfa was assessed by measuring the seeds primary root length (mm).

The plant root colonization phenotypes of three rainforests of *Paraburkholderia* [*Paraburkholderia tropica* J19-1 (BCC1950), *Paraburkholderia bannensis* J75-1 (BCC1915) and *Paraburkholderia* sp. J94 (BCC1909); Table 2] were evaluated using an *Arabidopsis thaliana* growth model as previously described [47]. *Escherichia coli* DH5*α* was used as a non-root colonizing negative control [47], the biopesticidal strains *Burkholderia ambifaria* BCC0191 [40] and metabolite-producing *Burkholderia gladioli* BCC1697 [42] were tested as known rhizosphere-colonizing *Burkholderia*, and *P. phytofirmans* PsJN included as a known plant growth-promoting *Paraburkholderia* species [11]. The strains were fluorescently labelled with a novel integrative vector, pCTX1_yeast_eGFP, which was based on the vector mini CTX [48]; full details on its construction are described in Petrova [49]. Visualization of the macroscopic root interaction phenotype was performed after 14 days of incubation using a Biospace Lab PhotonIMAGER Optima. For basic quantitative analysis of root growth in this experiment (*n*=1), the root length from the stem base to the longest root tip was measured (mm) and compared for the six plants in each test group: the control (no bacterial inoculation), *E. coli*, *Paraburkholderia* and *Burkholderia* inoculation groups. Comparison of the mean root length for the groups was performed using ANOVA and Tukey’s honestly significant difference testing.

The potential for endophytic interaction was examined by confocal microscopy as follows. *A. thaliana* seedlings inoculated with each strain were grown for 7 days on root-germination medium [1% (w/v) purified agar, 0.75% (w/v) sucrose and 0.25% (w/v) Murashige and Skoog basal salts medium; Duchefa Biochemie]. Seedlings were gently removed and washed three times with 15 ml sterile PBS. Each seedling was placed on a glass 76×26 mm microscopy slide, stained with 50 µl 0.01 mg ml^−1^ propidium iodide in PBS (Sigma) and covered with a borosilicate glass rectangular cover slip. The *A. thaliana* roots were imaged using a Zeiss LSM 710 inverted confocal microscope with the following settings: excitation with 488 nm laser (4.5%), emission channel 1(499–529 nm), emission channel 2(595–719 nm), 44 µm confocal pinhole and Plan-Apochromat 20×/0.8 M27 objective. ZEN 2009 microscopy software was used for image acquisition and processing.

## Results

### Isolation of bacteria from plant rhizosphere samples

A total of 123 bacterial isolates were obtained from a collection of 98 rhizosphere samples from plants growing on the Bornean rainforest floor. After initial screening by bacterial 16S rRNA and *recA* gene PCR-sequencing and rough identification by Nucleotide blast, a total of 89 *Burkholderia*/*Paraburkholderia*-related bacterial isolates were putatively identified, along with 34 isolates related to other bacterial genera. The non-*Burkholderia*/*Paraburkholderia* rainforest isolates were taxonomically diverse (Table 1) and composed of bacteria belonging to three phyla: *Actinomycetota*, *Bacillota* and *Pseudomonadota* (classes *Alphaproteobacteria*, *Betaproteobacteria* and *Gammaproteobacteria*). Isolates included representatives from the genera: *Curtobacterium* (14 isolates), *Leifsonia* (2 isolates), *Bacillus* (1 isolate), *Lactococcus* (2 isolates), *Enterococcus* (1 isolate), *Klebsiella* (5 isolates), *Pantoea* (4 isolates), *Enterobacter* (3 isolates), *Brucella* (1 isolate), *Caballeronia* (1 isolate) and *Pandoraea* (1 isolate) (Table 1). The preliminary isolate classification demonstrated that PCAT was only a semi-selective medium for *Burkholderia* and can enrich for *Paraburkholderia* and that the diversity of isolates enriched by PCAT should be checked carefully (Table 1).

To remove redundancy in the *Burkholderia/Paraburkholderia*-related rainforest isolates, basic strain genotyping via RAPD-PCR [24] was used (Fig. S2). RAPD profiling demonstrated that multiple isolates from the same soil sample were identical and those representative of diversity were selected for further analysis. The final rainforest collection comprised 95 isolates containing 15 *Burkholderia,* 46 *Paraburkholderia* and 34 other bacterial isolates (Table 1). This collection demonstrated that *Burkholderia* and *Paraburkholderia* were isolated from the rhizosphere across all sampling sites, except for their absence at site 6 (yielding *Enterobacter* and *Curtobacterium* species; Table 1). There was nothing specific about the location of site 6 (see Fig. S1) or the plants sampled that would explain the absence of *Burkholderia* and *Paraburkholderia*. Notably, site 5 yielded exclusively *Burkholderia* and *Paraburkholderia* species, and overall, sites 1 and 5 were dominated by *Paraburkholderia*, which represented >50% of the bacteria recovered from these sites (Fig. S3). From the final collection of 95 rainforest isolates, by using the preliminary 16S rRNA gene sequence analysis (see below), it was decided to genome sequence isolates that were putatively identified as belonging to the *Burkholderiaceae* (*Burkholderia*, *Paraburkholderia, Caballeronia* and *Pandoraea*). However, five *Burkholderia/Paraburkholderia* isolates and one *Pandoraea* isolate did not revive after cryopreservation and were removed (Table 2). Given the difficulty in classifying *Burkholderiaceae* using conventional phenotypic methods, the remaining 56 *Burkholderia/Paraburkholderia* isolates and one *Caballeronia* isolate were subjected to genome sequencing, and their taxonomy was assigned using a high-resolution genomic taxonomy approach [5] (Table 2).

### Phylogenetic and genomic assignment of *Burkholderiaceae* isolates

**Fig. 1.**
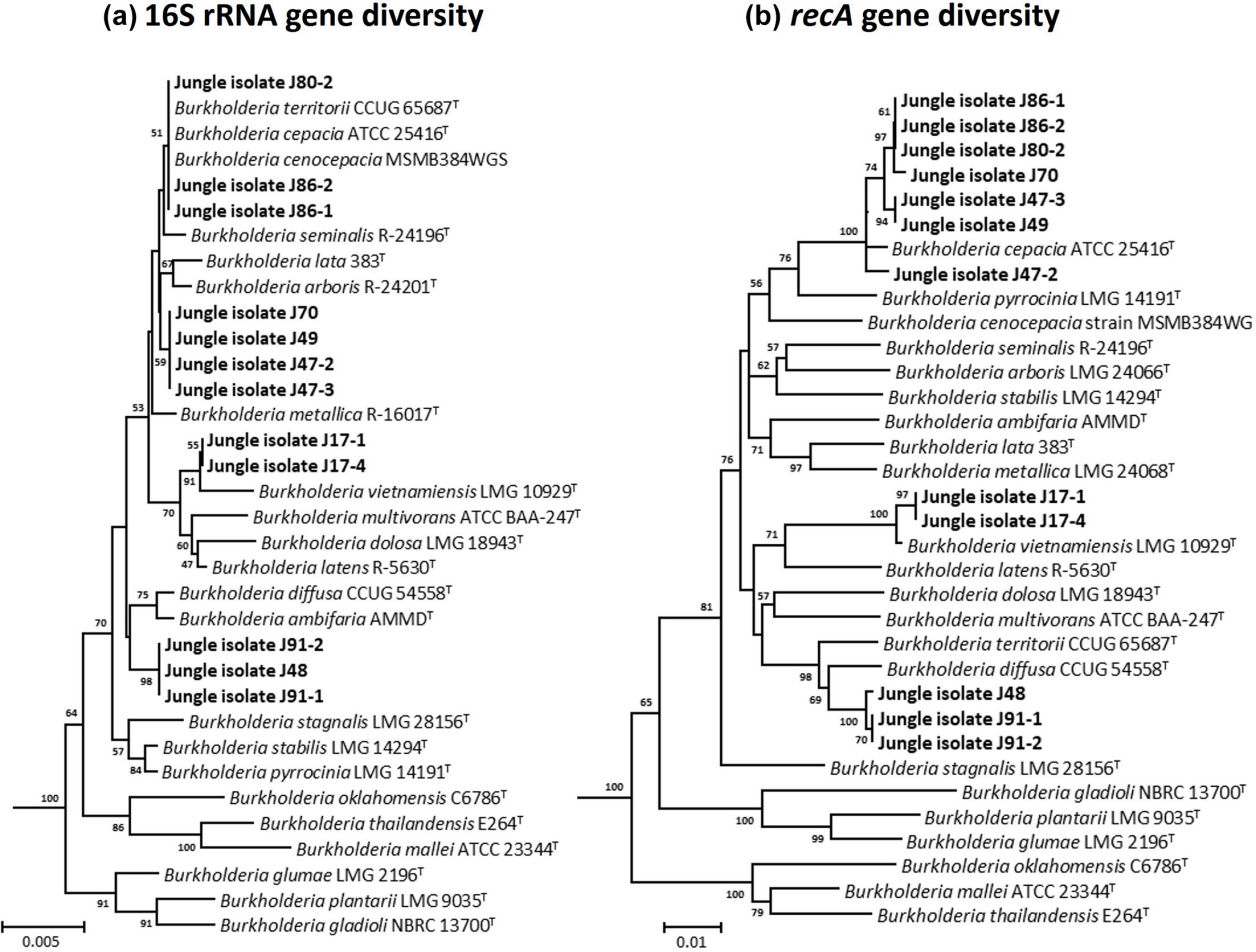
Phylogenetic placement of the rainforest rhizosphere *Burkholderia* isolates using the 16S rRNA and *recA* gene sequences. Clustering of the 16S rRNA gene (**a**) and *recA* gene (**b**) sequences is shown in relation to each other and the gene sequences from representative taxonomic type species. Trees were constructed using minimum evolution method, and evolutionary distances were computed using LogDet method. All positions containing gaps and missing data were eliminated, and there was a total of 1,497 and 1,066 positions in the final datasets, respectively. *P. tropica* LMG 22274 and *P. bannensis* NBRC 103871 16S rRNA and *recA* gene sequences were used, respectively, as outgroups. The percentage of replicate trees in which the associated taxa clustered together in the bootstrap test (1,000 replicates) are shown next to the branches. Rainforest isolates are identified in bold.

**Fig. 2.**
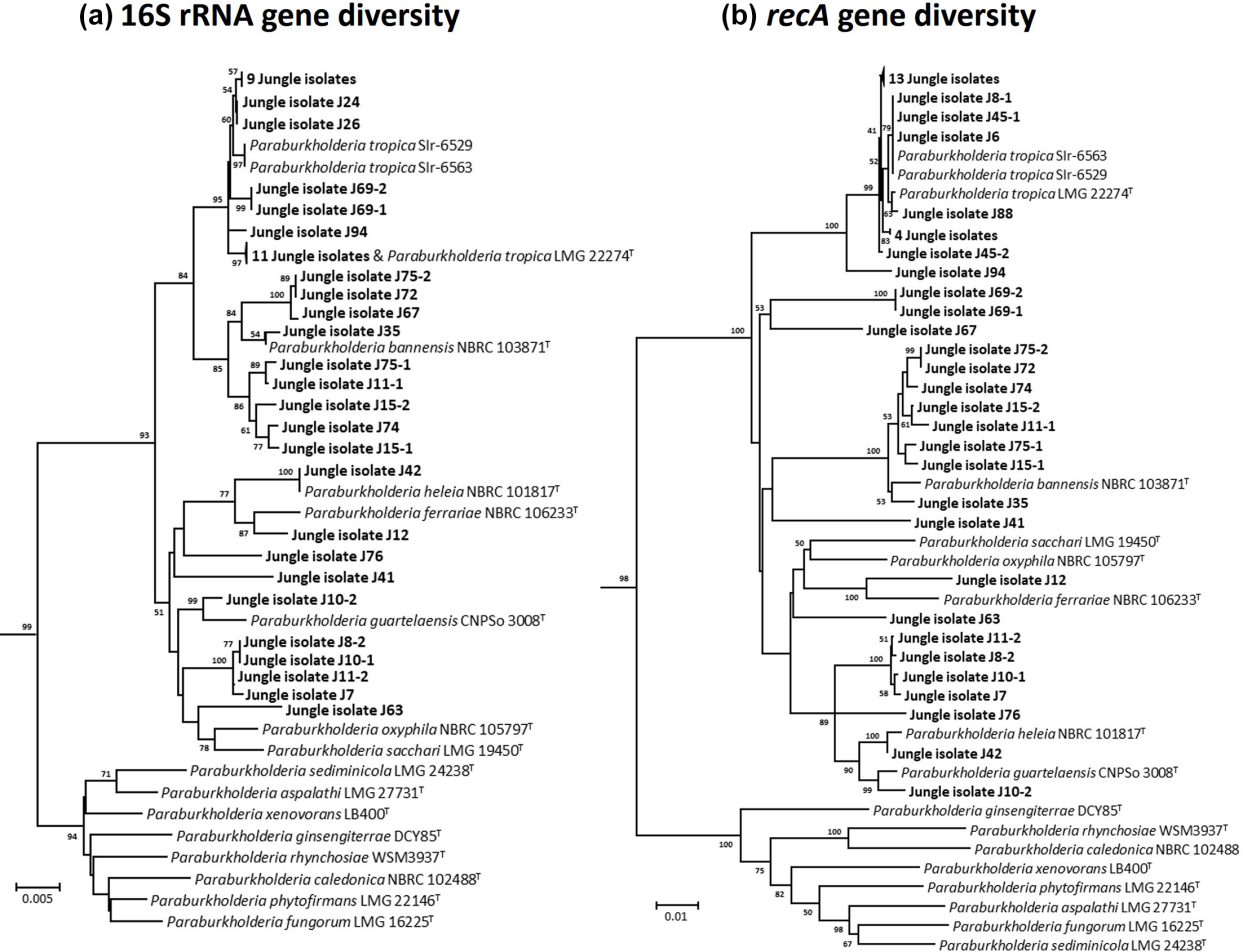
Phylogenetic analysis of the rainforest rhizosphere *Paraburkholderia* isolates using the 16S rRNA and *recA* gene sequences. Clustering of the 16S rRNA gene (**a**) and *recA* gene (**b**) sequences is shown in relation to each other and the gene sequences from representative taxonomic type species. Trees were constructed using minimum evolution method, and evolutionary distances were computed using LogDet method. All positions containing gaps and missing data were eliminated, and there was a total of 1,520 and 1,071 positions in the final datasets, respectively. Representative *Caballeronia* species and *Robbsia andropogonis* LMG 2129 16S rRNA and *recA* gene sequences, were respectively, used as outgroups. The percentage of replicate trees in which the associated taxa clustered together in the bootstrap test (1,000 replicates) are shown next to the branches. Rainforest isolates are identified in bold.

Phylogenetic analysis of 16S rRNA and *recA* genes extracted from rainforest isolate genomes was carried out and compared with genes from *Burkholderia* (Fig. 1), *Paraburkholderia* (Fig. 2) and *Caballeronia* (Fig. S4) reference genomes. Phylogenetic analysis of the 16S rRNA genes, from *Burkholderia* rainforest isolates grouped them into four distinct clusters: one cluster (J80-2, J86-1 and J86-2) with *B. cepacia, Burkholderia cenocepacia* and *Burkholderia territorii*, one cluster with *Burkholderia vietnamiensis* (J17-1 and J17-4) and two other novel groupings (Fig. 1a). Using the *recA* gene analysis (Fig. 1b), the *Burkholderia* rainforest isolates only split into three clusters: one large cluster grouping with *B. cepacia* (J80-2, J86-1, J86-2, J70, j47-3, j49 and j47-2), one cluster related to *B. vietnamiensis* (J17-1, J17-4) and one cluster associated with *Burkholderia diffusa* and *B. territorii* (J48, J91-1 and J91-2). Together, these analyses demonstrate that all *Burkholderia* recovered from the Bornean rainforest rhizosphere could be phylogenetically identified using single-gene markers as *B. cepacia, B. vietnamiensis* or associated with *B. diffusa* (Fig. 1).

Analysis of the *Paraburkholderia* 16S rRNA and *recA* genes followed a similar trend in that a different number of species-level groupings were obtained for each of the genes used (Fig. 2). Specifically, more potential *Paraburkholderia* species groupings were identified in phylogenetic trees made with the *recA* gene (12 species) than the 16S rRNA gene (nine species), although both trees were congruent in showing that most rainforest isolates belonged to either *P. tropica* or *P. bannensis* and that isolates J42 and J10-2 clustered with *Paraburkholderia heleia* and *Paraburkholderia guartelaensis*, respectively (Fig. 2). Rainforest isolate J12 also clustered closely with the type strain of *Paraburkholderia ferrariae* in both the *recA* and 16S rRNA gene trees (Fig. 2). The clustering of the remaining *Paraburkholderia* rainforest isolates demonstrated they were likely novel species by both single locus methods. One group of four closely related isolates (J7, J8-2, J10-1 and J11-2) formed a distinct novel cluster with both *recA* and 16S rRNA gene analyses. The one *Caballeronia* rainforest isolate, J97, was identified as a novel species by both *recA* and 16S rRNA gene analyses (Fig. S4).

**Fig. 3.**
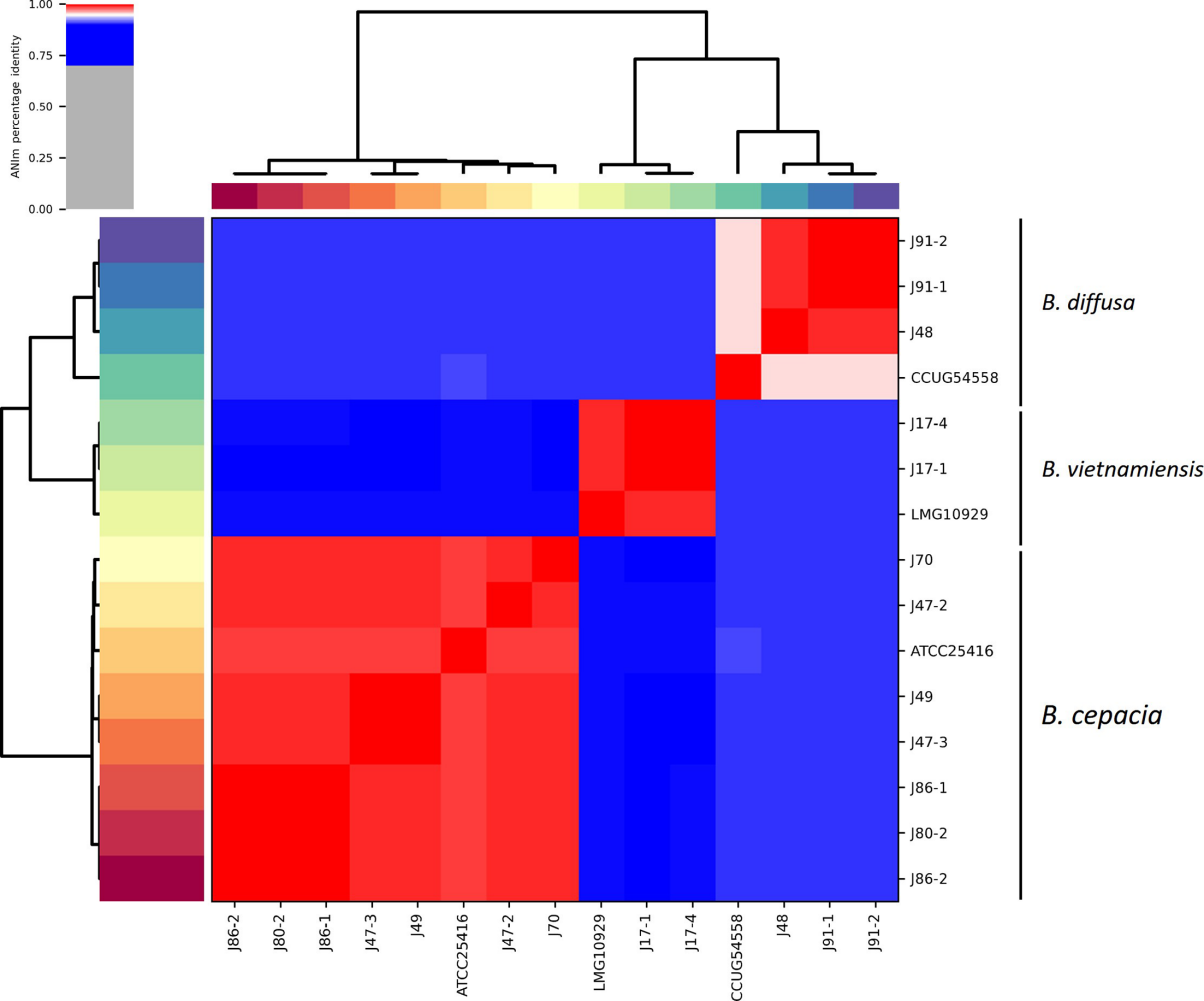
Genome sequence taxonomic placement of rainforest rhizosphere *Burkholderia* isolates inferred by ANI. A heatmap generated by the PyANI script is shown and indicates the degree of nucleotide-level similarity between *Burkholderia* isolates and their closest *Burkholderia* reference strains. Colour coding shows the degree of nucleotide similarity, with red areas indicating >95% ANI and darker shades of red indicating greater similarity. Blue indicates 70–95% ANI. Rainforest isolates identified by prefix J; CCUG54558*, B. diffusa* CCUG54558^T^; LMG10929*, B. vietnamiensis* LMG 10929^T^; ATCC25416*, B. cepacia* ATCC 25416^T^.

To increase the resolution of analysis, genomic classification [5] demonstrated the following for the 57 rainforest rhizosphere isolates. Sequenced genomes ranged in completeness from 98.2 to 99.9% and were assembled over 35 to 147 contigs (Table 2). Genome sequencing revealed that the genome size of *Paraburkholderia* rainforest isolates (mean=8.48 Mb±0.55 sd) was on average larger than those of *Burkholderia* (mean genome size=8.0 Mb±0.81 sd), and their mean %G+C content was lower with 64.4% (±0.74 sd) compared with 66.7% (±0.24 sd) for *Burkholderia* (Table 2). The genome size and %G+C for *Caballeronia* sp. J97 (BCC1952) was 7.8 Mb and 63.5%, respectively. To further assign the genomes to species level, ANI was also carried out on the assembled genomes. For the *Burkholderia* isolates, identification by genomic ANI (Fig. 3) correlated with the phylogenetic analysis of the *recA* gene (Fig. 1b), with all *Burkholderia* rainforest isolates grouping into three distinct species with >95% ANI to their respective taxonomic type strain genomes for *B. cepacia* (*n*=7), *B. vietnamiensis* (*n*=2) and *B. diffusa* (*n*=3). However, it should be noted that the ANI values for isolates J48, J91-1 and J91-2 were 95.6% ANI to *B. diffusa* type strain CCUG 54558^T^ genome and close to the threshold of 95% ANI used to differentiate bacterial species [34]. Furthermore, dDDH analysis conducted via the GGDC (with a species delineation threshold ≤70%) revealed that rainforest isolates J48, J91-1 and J91-2 had 63.5–63.7% dDDH similarity to the *B. diffusa* CCUG 54558^T^ genome (Table S1). This result suggested that isolates J48, J91-1 and J91-2 may be potentially classed as novel *Burkholderia* species, closely related to *B. diffusa*.

**Fig. 4.**
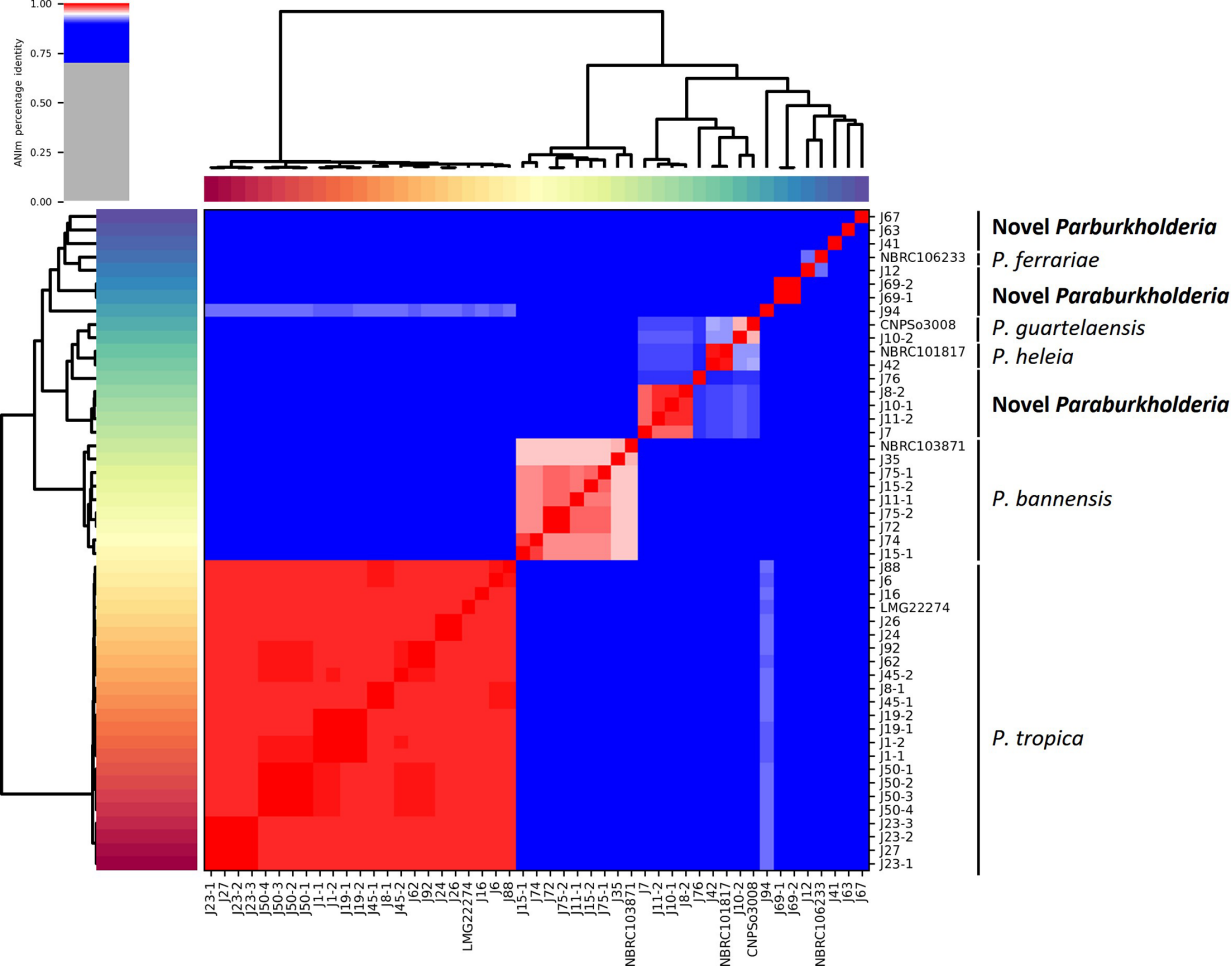
Genomic taxonomy of rainforest rhizosphere *Paraburkholderia* isolates inferred by ANI. A heatmap generated by the PyANI script is shown to indicate the degree of nucleotide-level similarity between *Paraburkholderia* isolates and their closest *Paraburkholderia* reference strains. The colour-coding indicates the degree of nucleotide similarity, with red areas indicating >95% ANI and darker shades of red indicating greater similarity. Blue indicates 70–95% ANI. Rainforest isolates identified by prefix J. NBRC106233*, P. ferrariae* NBRC 106233^T^; CNPSo3008, *P. guartelaensis* CNPSo 3008^T^; NBRC101817, *P. heleia* NBRC 101817^T^; NBRC103871, *P. bannensis* NBRC 103871^T^; LMG22274, *P. tropica* LMG 22274^T^.

The whole-genome identification by ANI of the 44 *Paraburkholderia* rainforest isolates (Fig. 4) was also broadly aligned to the analysis using *recA* (Fig. 2b), with most isolates being confirmed as *P. tropica* (*n*=22; 99.0–99.2% ANI to *P. tropica* LMG 22274^T^) and *P. bannensis* (*n*=8; 95.8–96.1% ANI to *P. bannensis* NBRC 103871^T^). However, dDDH analysis suggested that strains assigned by ANI to *P. bannensis* may potentially be novel *Paraburkholderia* closely related to *P. bannensis*, as dDDH values were 64.9–67.4% (Table S1). Rainforest isolates J42 and J10-2 were validated by ANI to be *P. heleia* and *P. guartelaensis*, with ANI values to type strains of 99.5% to *P. heleia* NBRC 101817^T^ and 96.3% to *P. guartelaensis* CNPSo3008^T^, respectively (Fig. 4). However, rainforest isolate J10-2 also had only 68.4% dDDH similarity to strain *P. guartelaensis* CNPSo3008^T^ and could be considered as a novel species (Table S1). The remaining 12 rainforest *Paraburkholderia* isolates split into eight novel species comprising a group of four isolates (J7, J8-2, J10-1 and J11-1), a group of two isolates (J69-1, J69-2) and six single-strain novel taxa by ANI (J12, J41, J63, J67, J-76 and J-94; Fig. 4, Table 1). Although isolate J12 clustered adjacent to *P. ferrariae* type strain NBRC106233^T^ by 16S rRNA and *recA* analysis (Fig. 2) and by ANI (Fig. 4), its ANI similarity value (91.9%) was below the 95% threshold (see Supplementary ANI Data File). The single *Caballeronia* rainforest isolate J97 was confirmed by genomic ANI and further phylogenomic analysis using TYGS to be a novel species (Figs S4 and S5, Table 2).

### Analysis of BGCs and assessment of antimicrobial activity

**Fig. 5.**
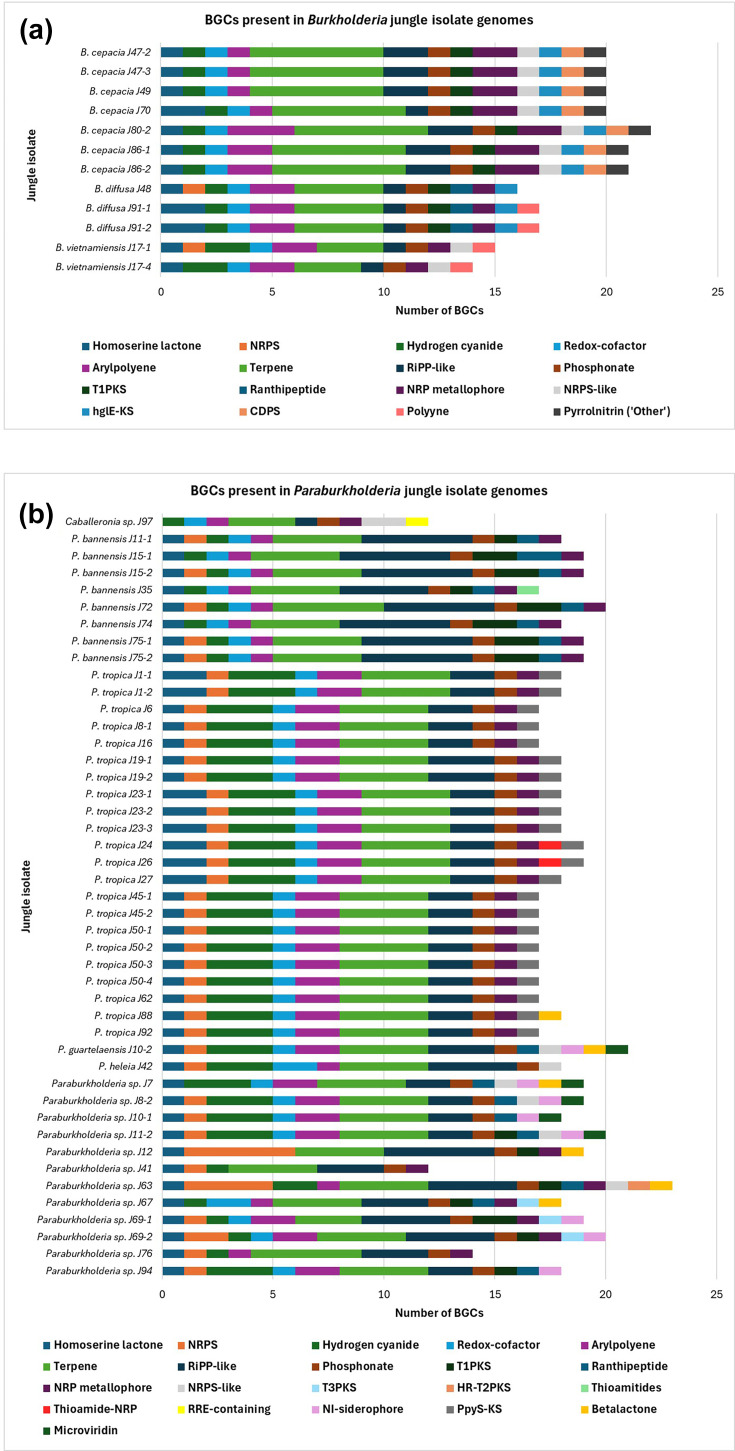
antiSMASH-predicted specialized metabolite BGC content of the rainforest *Burkholderia*, *Paraburkholderia* and Caballeronia sp. J97. The capacity and diversity of specialized metabolite BGCs within the rainforest *Burkholderia* (**a**) and *Paraburkholderia* and *Caballeronia* sp. J97 (**b**) is shown. Genome sequences were analysed using antiSMASH v7.1.0 [38] to broadly predict specialized metabolite BGC presence, with protein–protein blast used to identify the presence of polyyne encoding gene pathways. The cumulative number of BGCs is plotted for each isolate with the BGC class shown by the colour-coded key.

Following the prediction of specialized metabolite BGCs from the 57 sequenced rainforest isolate genomes, a total of 1,033 pathways were identified across the three sequenced *Burkholderiaceae* genera. The BGCs represented 25 known metabolite classes including terpenes, polyketide synthases (PKSs), non-ribosomal peptide synthetases (NRPSs), ribosomally synthesized and post-translationally modified peptide products (RiPP) and polyynes (Fig. 5). A small number (<1%) of BGCs, from *B. cepacia* genomes could not be assigned to a metabolite class by antiSMASH [38] and were described as ‘Other’ by the analysis (Fig. 5a). Further direct bioinformatic and homology analysis demonstrated that these *B. cepacia* BGCs were the same type and responsible for the production of pyrrolnitrin (Fig. 5a), as observed previously [40].

The *Paraburkholderia* genomes contained a greater diversity of BGC metabolite classes, with 20 distinct groups, compared to the 16 BGC groups found among the *Burkholderia* isolates. However, the average number of BGCs per genome was not significantly different, with 18.6 ± 2.6 in *Burkholderia* and 18.05±1.67 in *Paraburkholderia*, as determined by Welch’s t-test (t=0.695, df≈15.2, *P*>0.05). The most prevalent BGC classes for both genera were terpenes (23.2%), followed by RiPP-like (14.2%), hydrogen cyanide (11.3%) and aryl polyenes (9.1%), and collectively represented ~58% of the curated BGCs (Fig. 5b). Notably, *Paraburkholderia* also had an abundance of NRPS BGCs, whereas *Burkholderia* genomes contained NRP-metallophore and NRPS-like BGCs. Other BGCs uniquely detected in *Paraburkholderia* were type II and III PKSs, thioamitides, thioamide-linked NRPS, nickel-chelating siderophores (NI-siderophores), PPY-like pyrone KS, betalactones and microviridins (Fig. 5b). Heterocyst glycolipid synthase-like KS clusters, cyclodipeptide synthases, polyynes and pyrrolnitrin BGCs were found exclusively in *Burkholderia,* and a RiPP recognition element-containing cluster was uniquely identified in *Caballeronia* sp. J97 (Fig. 5b).

Under the growth conditions tested which utilized a glycerol carbon source-based growth medium (BSMG) known to stimulate metabolite production [20,43], antimicrobial bioactivity was observed exclusively among the *Burkholderia* rainforest isolates (Table 3, Fig. S6). No antimicrobial activity was detected for *Paraburkholderia* species or the single *Caballeronia* isolate (Table 3). Notably, *B. diffusa* (J91-1, J91-2) and *B. vietnamiensis* (J17-1, J17-4) exhibited antimicrobial activity against both Gram-positive bacteria (*S. aureus*) and fungi (*C. albicans*), whereas *B. cepacia* isolates demonstrated bioactivity only against *S. aureus* (Table 3). HPLC was used for direct screening of extracts from *Burkholderia* rainforest isolates grown on BSMG as a metabolite-inducing medium (Table S2, Fig. S6). Analysis confirmed that the rainforest isolates classified as *B. vietnamiensis* and *B. diffusa* produced cepacin [40], whereas those identified as *B. cepacia* produced pyrrolnitrin [50]. Both cepacin and pyrrolnitrin compounds have known activity against Gram-positive bacteria and fungi [40,50]. In addition, multiple unidentified HPLC peaks were also observed (especially for the *B. cepacia* rainforest isolates; up to seven peaks; Table S2) that could not be reconciled to known *Burkholderia* metabolites [43,51] and require further analysis to demonstrate if they are novel metabolites.

**Table 3.** Summary of potential plant growth-promoting genes and bioactivity for 57 rainforest rhizosphere *Burkholderiaceae* isolates

Rainforest isolate	BCC strain no.	Species	Phosphate solubilization (*pqq* genes)	Indole production (*iaa* genes)	Nitrogen fixation (*nif* genes)	Nitrogen fixation (fix genes)	Nitrogen fixation (other genes)	Bioactivity on BSMG at (pH 7, pH 5) against*		
								*S. aureus*	*P. carotovorum*	*C. albicans*
J1-1	BCC1935	*P. tropica*	*A, B, C, D, E*	*H*	*Q*	*A, B, I, J*	*hybE, nodI*	-, -	-, -	-, -
J1-2	BCC1936	*P. tropica*	*A, B, C, D, E*	*H*	*Q*	*A, B, I, J*	*hybE, nodI*	-, -	-, -	-, -
J6	BCC1918	*P. tropica*	*A, B, C, D, E*	*H*	*Q*	*A, B, I, J*	*hybE, nodI*	-, -	-, -	-, -
J7	BCC1912	*Paraburkholderia* sp.	*A, B, C, D, E*	*H*	*Q, U*	*A, B, I, J*	*nodI*	-, -	-, -	-, -
J8-1	BCC1949	*Paraburkholderia tropica*	*A, B, C, D, E*	*H*	*Q*	*A, B, I, J*	*hybE, nodI*	-, -	-, -	-, -
J8-2	BCC1943	*Paraburkholderia* sp.	*A, B, C, D, E*	*H*	*Q, U*	*A, B, I, J*	*nodI*	-, -	-, -	-, -
J10-1	BCC1954	*Paraburkholderia* sp.	*A, B, C, D, E*	*H*	*Q, U*	*A, B, I, J*	*nodI*	-, -	-, -	-, -
J10-2	BCC1942	*P. guartelaensis*	*A, B, C, D, E*	*H*	*Q*	*A, B, I, J*	*nodI*	-, -	-, -	-, -
J11-1	BCC1941	*P. bannensis*	*A, B, C, D, E*	*H*	*Q*	*A, B, I, J*	*nodI*	-, -	-, -	-, -
J11-2	BCC1940	*Paraburkholderia* sp.	*A, B, C, D, E*	*H*	*Q, U*	*A, B, I, J*	*nodI*	-, -	-, -	-, -
J12	BCC1953	*Paraburkholderia* sp.	–	*H*	*Q*	*A, I, J*	*nodI*	-, -	-, -	-, -
J15-1	BCC1939	*P. bannensis*	*A, B, C, D, E*	*H*	*Q*	*A, B, I, J*	*nodI*	-, -	-, -	-, -
J15-2	BCC1938	*P. bannensis*	*A, B, C, D, E*	*H*	*Q*	*A, B, I, J*	*nodI*	-, -	-, -	-, -
J16	BCC1937	*P. tropica*	*A, B, C, D, E*	*H*	*Q*	*A, B, I, J*	*hybE, nodI*	-, -	-, -	-, -
J17-1	BCC1956	*B. vietnamiensis*	*A, B, C, D, E*	*H*	*Q, A, B, D, E, H, K, N, T, V, W, X, Z*	*A, B, I, J*	*fdxB, hybE, nodI*	+, +	-, -	+, +
J17-4	BCC1955	*B. vietnamiensis*	*A, B, C, D, E*	*H*	*Q, A, B, D, E, H, K, N, T, V, W, X, Z*	*A, B, I, J*	*fdxB, hybE, nodI*	+, +	-, -	+, +
J19-1	BCC1950	*P. tropica*	*A, B, C, D, E*	*H*	*Q, J*	*A, B, I, J*	*hybE, nodI*	-, -	-, -	-, -
J19-2	BCC1951	*P. tropica*	*A, B, C, D, E*	*H*	*Q, J*	*A, B, I, J*	*hybE, nodI*	-, -	-, -	-, -
J23-1	BCC1948	*P. tropica*	*A, B, C, D, E*	*H*	*Q*	*A, B, I, J*	*hybE, nodI*	-, -	-, -	-, -
J23-2	BCC1947	*P. tropica*	*A, B, C, D, E*	*H*	*Q*	*A, B, I, J*	*hybE, nodI*	-, -	-, -	-, -
J23-3	BCC1946	*P. tropica*	*A, B, C, D, E*	*H*	*Q*	*A, B, I, J*	*hybE, nodI*	-, -	-, -	-, -
J24	BCC1934	*P. tropica*	*A, B, C, D, E*	*H*	*Q*	*A, B, I, J*	*hybE, nodI*	-, -	-, -	-, -
J26	BCC1933	*P. tropica*	*A, B, C, D, E*	*H*	*Q*	*A, B, I, J*	*hybE, nodI*	-, -	-, -	-, -
J27	BCC1945	*P. tropica*	*A, B, C, D, E*	*H*	*Q*	*A, B, I, J*	*hybE, nodI*	-, -	-, -	-, -
J35	BCC1932	*P. bannensis*	*A, B, C, D, E*	*H*	*Q*	*A, B, I, J*	*nodI*	-, -	-, -	-, -
J41	BCC1931	*Paraburkholderia* sp.	–	*H*	*Q*	*A, B, I, J*	*nodI*	-, -	-, -	-, -
J42	BCC1930	*P. heleia*	*A, B, C, D, E*	*H*	*Q*	*A, B, I, J*	*nodI*	-, -	-, -	-, -
J45-1	BCC1929	*P. tropica*	*A, B, C, D, E*	*H*	*Q*	*A, B, I, J*	*hybE, nodI*	-, -	-, -	-, -
J45-2	BCC1928	*P. tropica*	*A, B, C, D, E*	*H*	*Q*	*A, B, I, J*	*hybE, nodI*	-, -	-, -	-, -
J47-2	BCC1959	*B. cepacia*	*A, B, C, D, E*	*H*	*Q*	*A, B, I, J*	*nodI*	-, +	-, -	-, -
J47-3	BCC1958	*B. cepacia*	*A, B, C, D, E*	*H*	*Q*	*A, B, I, J*	*nodI*	-, +	-, -	-, -
J48	BCC1957	*B. diffusa*	*A, B, C, D, E*	*H*	*Q*	*A, B, I, J*	*nodI*	-, +	-, -	-, -
J49	BCC1967	*B. cepacia*	*A, B, C, D, E*	*H*	*Q*	*A, B, I, J*	*nodI*	-, +	-, -	-, -
J50-1	BCC1927	*P. tropica*	*A, B, C, D, E*	*H*	*Q, A, B, D, E, H, K, N, T, V, W, X, Z*	*A, B, I, J*	*fdxB, hybE, nodI, cowN*	-, -	-, -	-, -
J50-2	BCC1926	*P. tropica*	*A, B, C, D, E*	*H*	*Q, A, B, D, E, H, K, N, T, V, W, X, Z*	*A, B, I, J*	*fdxB, hybE, nodI, cowN*	-, -	-, -	-, -
J50-3	BCC1925	*P. tropica*	*A, B, C, D, E*	*H*	*Q, A, B, D, E, H, K, N, T, V, W, X, Z*	*A, B, I, J*	*fdxB, hybE, nodI, cowN*	-, -	-, -	-, -
J50-4	BCC1924	*P. tropica*	*A, B, C, D, E*	*H*	*Q, A, B, D, E, H, K, N, T, V, W, X, Z*	*A, B, I, J*	*fdxB, hybE, nodI, cowN*	-, -	-, -	-, -
J62	BCC1923	*P. tropica*	*A, B, C, D, E*	*H*	*Q*	*A, B, I, J*	*hybE, nodI*	-, -	-, -	-, -
J63	BCC1922	*Paraburkholderia* sp.	–	*H*	*Q, U*	*A, B, I, J, H*	*nodI*	-, -	-, -	-, -
J67	BCC1921	*Paraburkholderia* sp.	*A, B, C, D, E*	*H*	*Q*	*A, B, I, J*	*nodI*	-, -	-, -	-, -
J69-1	BCC1920	*Paraburkholderia* sp.	*A, B, C, D, E*	*H*	*Q*	*A, B, I, J*	*nodI*	-, -	-, -	-, -
J69-2	BCC1919	*Paraburkholderia* sp.	*A, B, C, D, E*	*H*	*Q*	*A, B, I, J*	*nodI*	-, -	-, -	-, -
J70	BCC1960	*B. cepacia*	*A, B, C, D, E*	*H*	*Q*	*A, B, I, J*	*nodI*	-, +	-, -	-, -
J72	BCC1917	*P. bannensis*	*A, B, C, D, E*	*H*	*Q*	*A, B, I, J*	*nodI*	-, -	-, -	-, -
J74	BCC1916	*P. bannensis*	*A, B, C, D, E*	*H*	*Q*	*A, B, I, J*	*nodI*	-, -	-, -	-, -
J75-1	BCC1915	*P. bannensis*	*A, B, C, D, E*	*H*	*Q*	*A, B, I, J*	*nodI*	-, -	-, -	-, -
J75-2	BCC1914	*P. bannensis*	*A, B, C, D, E*	*H*	*Q*	*A, B, I, J*	*nodI*	-, -	-, -	-, -
J76	BCC1913	*Paraburkholderia* sp.	–	*H*	*Q, U*	*A, I, J*	*nodI*	-, -	-, -	-, -
J80-2	BCC1963	*B. cepacia*	*A, B, C, D, E*	*H*	*Q*	*A, B, I, J*	*nodI*	-, +	-, -	-, -
J86-1	BCC1962	*B. cepacia*	*A, B, C, D, E*	*H*	*Q*	*A, B, I, J*	*nodI*	-, +	-, -	-, -
J86-2	BCC1961	*B. cepacia*	*A, B, C, D, E*	*H*	*Q*	*A, B, I, J*	*nodI*	-, +	-, -	-, -
J88	BCC1911	*P. tropica*	*A, B, C, D, E*	*H*	*Q*	*A, B, I, J*	*hybE, nodI*	-, -	-, -	-, -
J91-1	BCC1965	*B. diffusa*	*A, B, C, D, E*	*H*	*Q*	*A, B, I, J*	*nodI*	+, +	-, -	+, -
J91-2	BCC1964	*B. diffusa*	*A, B, C, D, E*	*H*	*Q*	*A, B, I, J*	*nodI*	+, +	-, -	+, -
J92	BCC1910	*P. tropica*	*A, B, C, D, E*	*H*	*Q*	*A, B, I, J*	*hybE, nodI*	-, -	-, -	-, -
J94	BCC1909	*Paraburkholderia* sp.	*A, B, C, D, E*	*H*	*Q*	*A, B, I, J*	*hybE, nodI*	-, -	-, -	-, -
J97	BCC1952	*Caballeronia* sp.	*A, B, C, D, E*	–	*Q*	*A, B, I, J*	*nodI*	-, -	-, -	-, -

*+, detectable bioactivity against the susceptibility testing micro-organism; -, no detectable activity against the susceptibility testing micro-organism.

BCC strain numbers refer to the number given in the Cardiff University *Burkholderia* Culture Collection.

### Genomic PGP properties

**Fig. 6.**
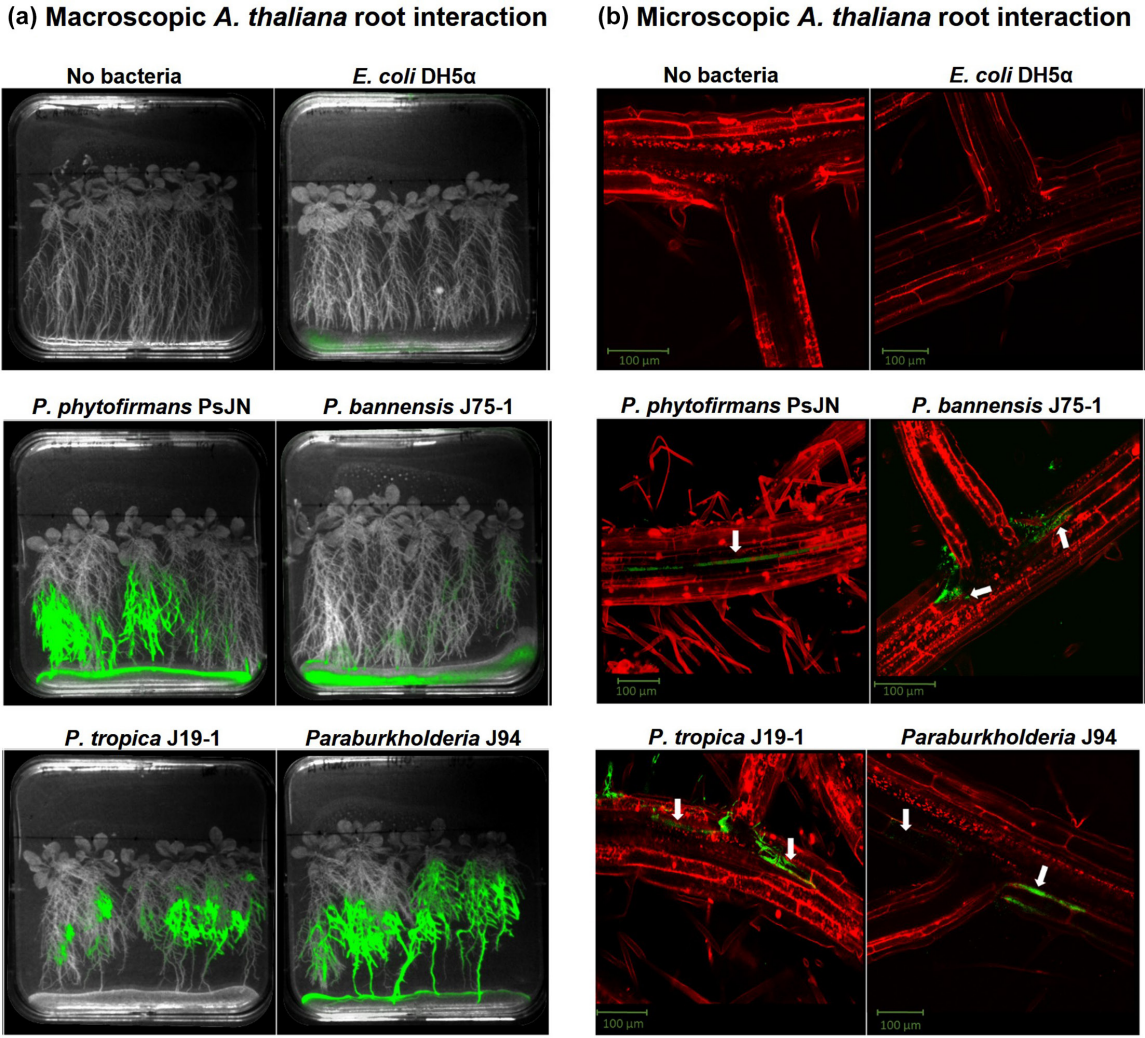
*Paraburkholderia* demonstrate active root colonization and endophytic interactions in the *A. thaliana* rhizosphere interaction model. (**a**) For macroscopic plant interaction analysis, *A. thaliana* seedlings were grown vertically in the root colonization model (2 weeks), and the fluorescently labelled *Paraburkholderia* suspension in soft agar was applied to the lower edge of the plate without touching the roots. After another 7-day vertical incubation post-inoculation, the plates were imaged using a Biospace Labs PhotonIMAGER Optima at excitation wavelength 488 nm and emission wavelength 522 nm. The images are representative of eight different plates examined in four independent experiments. (**b**) To understand the cellular plant interaction phenotype of the *Paraburkholderia*, confocal microscopy was performed as follows. Seven days post the bacterial soft agar inoculation [panel (a)], individual seedlings were removed from the plates, washed with PBS, stained with propidium iodide, immediately placed on a microscope slide with a cover slip and imaged by inverted confocal microscopy. The images are representative of ten different fields examined for each bacteria–plant interaction. Vasculature-associated endophytic colonization is indicated by white arrows.

Bioinformatic analysis of the 57 genomes demonstrated that all the rainforest isolates had the genetic potential to promote plant growth by enhancing phosphate solubilization, auxin production or nitrogen fixation. The majority of the isolates (*n*=53) were predicted to be capable of solubilizing phosphate, supported by the presence of the *pqq* gene cluster (Table 3), and was also confirmed by antiSMASH under the ‘Redox-cofactor’ BGC category (Fig. 6). Only isolates J12, J41, J63 and J76 lacked the *pqqABCD* gene cluster, and notably, these were all individual representatives of novel *Paraburkholderia* species (Table 3, Figs 2 and 4). The presence of the complete *pqqABCDE* gene cluster codes for the biosynthesis of the cofactor pyrroloquinoline quinone (PQQ), essential for PQQ-dependent glucose dehydrogenase activity. This enzyme facilitates the transformation of glucose to gluconic acid, a key step in the oxidative pathway of glucose metabolism leading to enhanced inorganic P solubilization [52]. To validate these genomic predictions, rainforest isolates *P. tropica* J19-1, *P. bannensis* J75-1 and J75-2, all of which possess the complete *pqq* cluster (Table 3), were tested for phosphate-solubilizing activity using the NBRIP agar plate assay [45]. All three rainforest *Paraburkholderia* demonstrated consistent formation of clear zones on the growth medium and possessed an equivalent phosphate-solubilizing activity to the control *P. phytofirmans* strain PsJN (LMG 22146^T^; Table S3).

The essential genes (*nif*, *fix* and *fdx*) involved in nitrogen fixation [53] were evident in all rainforest *Burkholderia* and *Paraburkholderia* isolates (Table 3). However, only six isolates encoded complete *nif* operons along with both *fix* and *fdx* genes (Table 3). These isolates belonged to *B. vietnamiensis* (J17-1, J17-4) and *P. tropica* (J50-1, J50-2, J50-3 and J50-4), both of which are species known to fix atmospheric nitrogen [54,55]. However, the majority of the remaining *P. tropica* rainforest isolates (18 of 22) did not possess a complete set of nitrogen fixation genes and only contained *nifQ* or *nifJ* along with several *fix* genes (Table 3). Three rainforest isolates, *P. bannensis* J75-1, *P. bannensis* J75-2 and *P. tropica* J19-1, were able to grow in the absence of a nitrogen source on a minimal salts growth medium with glycerol as a carbon source (modified BSMG) [20] (Table S3). Of particular interest was the conserved presence of the nod factor gene, *nodI*, in all *Burkholderiaceae* rainforest isolates (Table 3). Nodulation factors are typically produced by symbiotic nitrogen-fixing bacteria and play a key role in the signalling during root nodule formation in the host plants [12].

The rainforest isolate genomes were also screened for the *iaaH* gene involved in the biosynthesis of the plant hormone IAA (or auxin) [56]. Specifically, *iaaH* encodes the enzyme indole-3-acetamide (IAM) hydrolase, responsible for the conversion of IAM to IAA. All analysed *Burkholderia* and *Paraburkholderia* genomes contained the *iaaH* gene, and only *Caballeronia* rainforest isolate J97 lacked *iaaH* (Table 3). The potential of *Paraburkholderia* strains carrying *iaaH* to synthesize IAA was also confirmed by the presence of detectable indoles by using a modified Salkowski’s reagent assay (Table S3). Interestingly, *P. tropica* J19-1 produced levels of IAA comparable to the *P. tropica* type strain LMG 22274^T^ and stimulated alfalfa root development to an equivalent extent (Table S3).

### *Paraburkholderia* demonstrate a highly active, endophytic root colonization phenotype

To further expand the ecological understanding of the rainforest *Paraburkholderia,* the plant root interaction phenotypes of three strains, *P. tropica* J19-1, *P. bannensis* J75-1 and *Paraburkholderia* sp. J94 (Table 2), were investigated using an *A. thaliana* rhizosphere colonization model [47]. These three strains had also previously been shown to be capable of heterologous expression and production of the plant-protective biopesticidal *Burkholderia* metabolite caryoynencin [57]. All three rainforest *Paraburkholderia* and the *P. phytofirmans* PsJN-positive control demonstrated an active *A. thaliana* root colonization phenotype when GFP-labelled strains were macroscopically visualized at the rhizosphere (Fig. 6a). The root interactions of the *Paraburkholderia* were directly compared with *E. coli* DH5*α* as a negative control (Fig. 6a) and *B. gladioli* BCC1697 [42] and *B. ambifaria* BCC0191 [40] as known rhizosphere colonizers which both possess protective phenotypes against damping-off disease [40,42] (Fig. S7A). Interestingly, while both *Burkholderia* strains eventually colonized the root system, *A. thaliana* root growth in their presence was significantly delayed (*P*<0.001) compared to the no bacteria control and *Paraburkholderia* treatment groups, as well as the *E. coli-*inoculated group (*P*<0.05; Fig. S8). For all the *Paraburkholderia* strains evaluated, the *A. thaliana* roots grew well and readily made contact with the bacterial inoculum encased in the soft agar at the bottom of the growth plate (Fig. S7A).

The active *Paraburkholderia* root colonization phenotype (Fig. 6a) was further evaluated at the microscopic level using confocal analysis of *A. thaliana* root sections (Fig. 6b). No evidence of root colonization was observed for the *E. coli* DH5*α* negative control (Fig. 6b). In contrast, the *Paraburkholderia* showed extensive evidence of endophytic root colonization and spread within *A. thaliana. Paraburkholderia* sp*.* BCC1909, *P. bannensis* BCC1915 and *P. tropica* BCC1950 were localized in the cytoplasm of the plant cells in a comparable manner to the *P. phytofirmans* PsJN endophytic positive control [11] which also colonized the internal vasculature of the root (Fig. 6b). Interestingly, both *B. gladioli* BCC1697 and *B. ambifaria* BCC0191 were localized to sites where the lateral roots emerged from the main tap root, breaking through the outer two cell layers of epidermis and cortex, but not spreading further within the *A. thaliana* root system (Fig. S7B).

## Discussion

Our genomic taxonomy-based survey of the Bornean rainforest rhizosphere has shown it is a rich reservoir of novel *Burkholderiaceae* taxa, especially *Paraburkholderia* including those that are known to mediate PGP, and novel species of as yet uncharacterized function. Although enrichment from the rhizosphere was performed using selective media known to promote the recovery of *Burkholderia* species [21], these were limited to *B. cepacia, B. vietnamiensis* and *B. diffusa*-like strains, and the resulting collection was dominated by *Paraburkholderia* (Tables 1 and 2). *P. tropica* and *P. bannensis* were the prevalent known species; however, a key highlight of the survey was the isolation of eight putative novel *Paraburkholderia* genomic taxa. Genomic analysis demonstrated that the *Paraburkholderia* encoded a broad range of BGCs and plant-promoting traits, yet observed antimicrobial activity and the production of known specialized metabolites was exclusive to the *Burkholderia* in the collection. Further *in vitro* and *in planta* analysis demonstrated that rainforest *Paraburkholderia* were capable of phosphate solubilization, auxin production and active endophytic root colonization. The survey highlights the potential of the tropical rainforest rhizosphere as a significant reservoir of *Burkholderiaceae* taxa that help support the ecological basis of rhizosphere health in the rainforest microbiome and are also suitable for harnessing in future sustainable agriculture.

### Antimicrobial activity and capacity for specialized metabolite synthesis

In this study, *in vitro* antimicrobial activity was observed exclusively in the *Burkholderia* species rainforest isolates, consistent with the published literature that highlights the genus as a rich source of natural products [6]. The ability to produce cepacin (Fig. S6), a potent biopesticide with activity against crop-pathogenic oomycetes [40], was associated with the *B. diffusa* and *B. vietnamiensis* rainforest isolates, while the antifungal activity seen in *B. cepacia* recovered could be explained by the presence of pyrrolnitrin (Table 3, Fig. S6). The presence of these antimicrobial *Burkholderia* metabolites also correlated to the genomically predicted BGCs for these rainforest isolates (Fig. 5a). Interestingly, despite their extensive genomic potential to encode specialized metabolite BGCs (Fig. 5b), the *Paraburkholderia* rainforest isolates exhibited no detectable *in vitro* antimicrobial activity (Table 3). This lack of detectable *in vitro* antimicrobial activity may be attributable to the growth conditions employed, despite the use of a glycerol-based screening medium known to induce the biosynthesis of multiple *Burkholderia* metabolites [20,43]. Dahal *et al.* [58] recently described two new, single-isolate species, *Paraburkholderia antibiotica* and *Paraburkholderia polaris*, both of which possessed antimicrobial activity. However, their screening method employed large volume liquid culture (R2A medium) followed by solvent extraction of the culture supernatant, which was concentrated and screened via a disc diffusion antimicrobial assay to reveal the anti-Gram-negative activity which was not further characterized at the metabolite level [58]. Given the latter result [58] and the rich diversity of genomic BGCs (Fig. 5b), future work should employ a broad range of different growth conditions and antimicrobial screening approaches [59] to determine whether novel bioactive compounds can be isolated from the diversity of rainforest *Paraburkholderia* characterized herein.

### *P. tropica* as the dominant culturable *Paraburkholderia* species capable of N fixation

Within this survey, *P. tropica* was the most common species recovered from the rainforest rhizosphere with a total of 26 isolates identified by *recA* analysis out of the 95 isolates, of which 22 were confirmed by genome sequence ANI (Table 1). *P. tropica* has been widely studied in relation to its nitrogen fixation capabilities, PGP properties and interactions with crops such as sugarcane, oil palm and tomato [60]. It is also a non-symbiotic nitrogen-fixing species with the ability to fix nitrogen outside of nodule formation as free-living species [60]. Within the collection of 22 *P. tropica* rainforest isolates that were genome sequenced, only four (J50-1 to J50-4; Table 3) encoded the full complement of genes required for nitrogen fixation including *nifV* as the homocitrate synthase essential for nitrogenase function in free-living conditions [60]. This revealed that the genomic potential for free-living nitrogen fixation was not a universal trait for the diversity of *P. tropica* isolates recovered in this study. Madhaiyan *et al.* [61] performed a survey of endophytic bacteria in Singapore and found *P. tropica* to be the most common species recovered (12 of 19 isolates). All the *P. tropica* isolates were recovered from oil palm and were not associated with *Acacia* as the other plant species sampled in the research [61]. Given that multiple oil palm plantations flank the Lower Kinabatangan River Wildlife Sanctuary, it would be valuable to investigate the extent to which these commercial crops sequester *Paraburkholderia* species from the rainforest soils and whether the subsequent rhizosphere interactions are supportive of their growth.

### Rainforest *Paraburkholderia* are active endophytic root colonizers

Multiple studies have demonstrated endophytic plant root interaction to be a key trait of *Paraburkholderia* species [60,62] since the early discoveries of *P. phymatum* as a nodulating nitrogen-fixing species [12] and demonstration of endophytic spread of *P. phytofirmans* PsJN within the grapevine (*Vitis vinifera* L.) root system [63]. For all but two of our sampling sites, homogenized root sections of rainforest floor plants formed the environmental basis from which bacteria were grown (Table 1). Hence, it was not surprising that when three selected rainforest *Paraburkholderia* strains were investigated for rhizosphere interaction within an *A. thaliana* [47] model, positive root colonization phenotypes were observed (Fig. 6a). Few *Paraburkholderia* species have been examined at the microscopic level in terms of their root colonization phenotypes. *P. phytofirmans* PsJN has been the most widely studied as a model endophytic species [62], with GFP-labelled derivatives shown to colonize the root tips, lateral root junctions and xylem of *V. vinifera* L. [63] and the root vasculature of *A. thaliana* [64]. *Paraburkholderia bryophila* Ha185 has also been characterized as a phosphate-solubilizing species and shown via confocal microscopy to colonize endophytically within ryegrass roots [65]. Here, using a novel integrative vector, pCTX1_yeast_eGFP, three rainforest *Paraburkholderia* strains were successfully labelled with GFP and shown to form extensive endophytic interactions with *A. thaliana* (Fig. 6b). The *Paraburkholderia* endophytic interactions also showed greater spread throughout the *A. thaliana* root vasculature (Fig. 6b) compared to the two biopesticidal *Burkholderia* tested, *B. gladioli* [42] and *B. ambifaria* [40]. The two *Burkholderia* strains were localized towards the lateral root junctions (Fig. S7B), perhaps suggesting a more opportunistic colonization based on access to the emerging lateral root-penetrated epidermal layer. Since heterologous expression of the biopesticidal *Burkholderia* polyyne caryoynencin was also successfully achieved in the same three *Paraburkholderia* strains in a previous study [57], this endophytic ability to move through plant root systems could be harnessed for future biotechnological delivery of other protective or growth-promoting gene clusters.

In summary, the Bornean rainforest rhizosphere is rich in cultivatable *Burkholderia* and *Paraburkholderia* species, with the latter having the greatest presence of novel species diversity within the collection recovered herein. Genomic taxonomy proved essential in distinguishing the *Burkholderiaceae* species, with multiple novel taxa separated by ANI from known species, and strains which pushed the upper threshold of the 95% species cut-off [5,34] in the context of the *B. diffusa*-like isolates. While the ecological role of *Burkholderiaceae* in natural tropical habitats needs further understanding, it is clear from the genomic and phenotypic traits they possess that they can be harnessed for multiple applications in biotechnology. The *Paraburkholderia* in particular show promise as plant beneficial biotechnological agents that are not encountered as pathogens and, as shown previously, are highly restricted in growth above 37 °C, yet capable of expressing complex BGCs [57]. Overall, our study has considerably expanded the genomic resources and cultivated isolates of *Paraburkholderia* available for further study in the context of their natural product biosynthesis and PGP.

## Supplementary material

10.1099/mgen.0.001720 Uncited Supplementary Material 1.

10.1099/mgen.0.001720 Uncited Supplementary Material 2.

## Abbreviations

ANI: average nucleotide identity
BGC: biosynthetic gene cluster
BSMG: basal salt medium with glycerol
CDS: coding sequence
DCM: dichloromethane
dDDH: digital DNA–DNA hybridization
DGFC: Danau Girang Field Centre
GGDC: Genome-to-Genome Distance Calculator
IAA: indole-3-acetic acid
IAM: indole-3-acetamide
NBRIP: National Botanical Research Institute’s phosphate solubilization agar
NRPS: non-ribosomal peptide synthetase
PCAT: *Pseudomonas cepacia* azelaic acid tryptamine
PGP: plant growth promotion
PKS: polyketide synthase
PQQ: pyrroloquinoline quinone
RAPD: random amplified polymorphic DNA
RiPP: ribosomally synthesized and post-translationally modified peptide products
RT: room temperature
TSB: tryptone soya broth
TYGS: Type (Strain) Genome Server
YM: yeast malt medium
