# Supplementary material for: Genomic diversity of culturable Paraburkholderia and Burkholderia species isolated from Bornean rainforest rhizosphere
Source: Microb Genom. 2026 May 15;12(5):001720. doi: 10.1099/mgen.0.001720 (PMC13178297; doi:10.1099/mgen.0.001720)
Supplement: Uncited Supplementary Material 1. [file mgen-12-01720-s001.pdf]

Supplementary Data

Genomic diversity of culturable *Paraburkholderia* and *Burkholderia* species isolated from Bornean rainforest rhizosphere

Author Names:

Amal Alswat[a,c], Gordon Webster[a], Alex J. Mullins[a,d], Yoana D. Petrova[a], Benjamin J. Davies[a], Angharad R. Jones[a], James A. H. Murray[a], Andrew J. Weightman[a], Benoit Goossens[a,b] and Eshwar Mahenthiralingam[a]

Affiliations

[a] School of Biosciences, Cardiff University, Cardiff, Wales, CF10 3AX, UK.

[b] Danau Girang Field Centre, c/o Sabah Wildlife Department, Wisma MUIS, Kota Kinabalu, Sabah, Malaysia

[c] Present address: Department of Biotechnology, College of Science, Taif University, P.O. Box 11099, Taif 21944, Saudi Arabia

[d] Present address: Department of Chemistry, University of Warwick, Coventry, England, CV4 7AL, UK.

Contents:

Supplementary Figures

1. Supplementary Figure S1. Location of rainforest rhizosphere soil sampling in Sabah, Borneo, Malaysia.

2. Supplementary Figure S2. Example of the RAPD-PCR screening used to remove redundancy in the collection of rainforest *Burkholderia* and *Paraburkholderia* isolates.

3. Supplementary Figure S3. Diversity of rainforest rhizosphere *Burkholderiaceae* isolates from nine different sampling sites (see Supplementary Figure S1) in the DGFC.

4. Supplementary Figure S4. Phylogenetic placement of the rainforest rhizosphere *Caballeronia* isolate J97 using the 16S rRNA and *recA* gene sequences.

5. Supplementary Figure S5. Genomic taxonomy of rainforest rhizosphere *Caballeronia* isolate J97.

6. Supplementary Figure S6. Antimicrobial bioactivity and metabolite characterization of selected rainforest *Burkholderia*.

7. Supplementary Figure S7. *Burkholderia* macroscopic and microscopic root colonisation interactions in the *A. thaliana* rhizosphere interaction model.

8. Supplementary Figure S8. The presence of *Burkholderia* significantly delays *A. thaliana* root growth within the rhizosphere interaction model

Supplementary Tables

1. Supplementary Table S1. Selected examples of pairwise ANI and *in silico* DDH values between rainforest isolates and their closest representative type strain.

2. Supplementary Table S2. HPLC analysis of *Burkholderia* rainforest isolates demonstrating the presence of known and unidentified specialised metabolites.

3. Supplementary Table S3. Plant growth promotion properties of selected *Paraburkholderia* rainforest isolates.

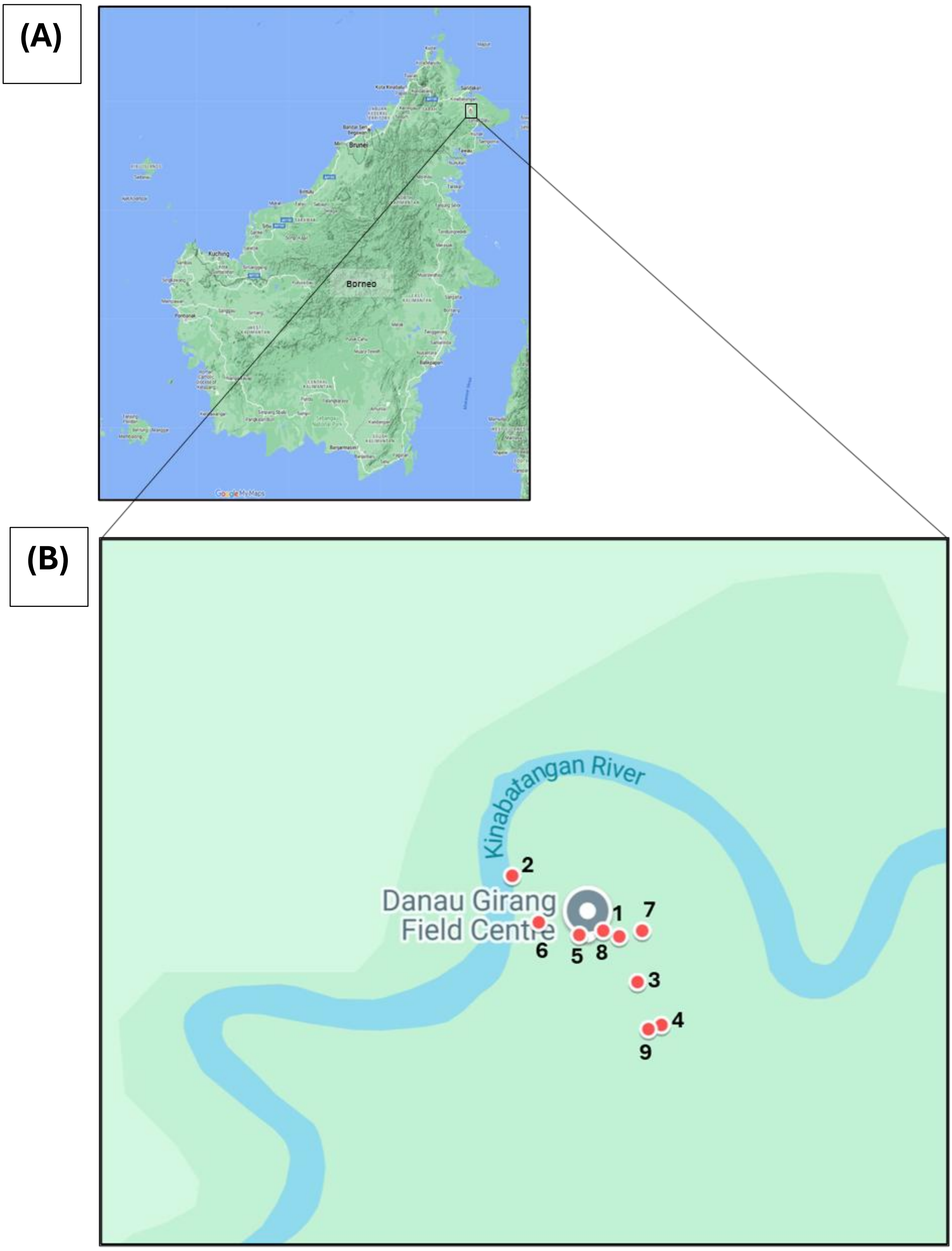

Supplementary Figure S1. Location of rainforest rhizosphere soil sampling in Sabah, Borneo, Malaysia. (A) The location of the Danau Girang Field Centre (DGFC) situated on the Kinabatangan River on the north-east coast of Borneo in the Malaysian State of Sabah. **(B)** Map showing the DGFC field sampling sites labelled 1 to 9. Map of Borneo generated by Google Maps (2024).

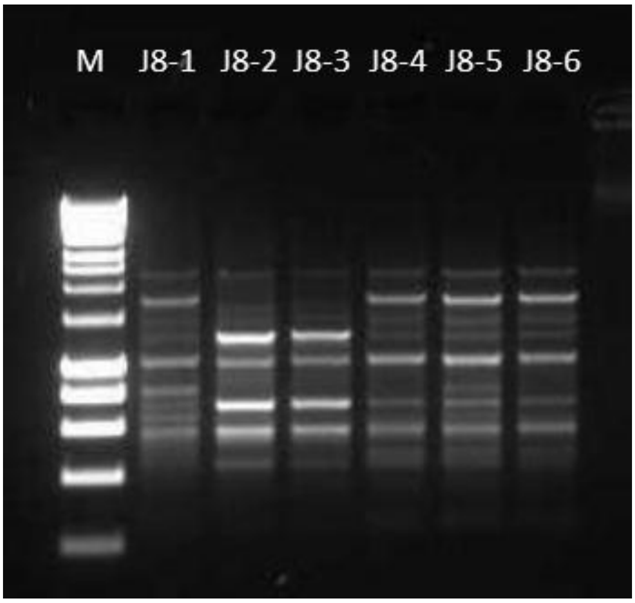

Supplementary Figure S2. Example of the RAPD-PCR screening used to remove redundancy in the collection of rainforest *Burkholderia* and *Paraburkholderia* isolates. The example shown demonstrates two distinct RAPD profiles obtained from six *Burkholderia*/*Paraburkholderia*-related isolates from rhizosphere soil sample J8 (sampling site 1). Isolates J8-1 and J8-2 were added to the final rainforest isolate collection. M, HyperLadder™ 1kb Marker (Bioline).

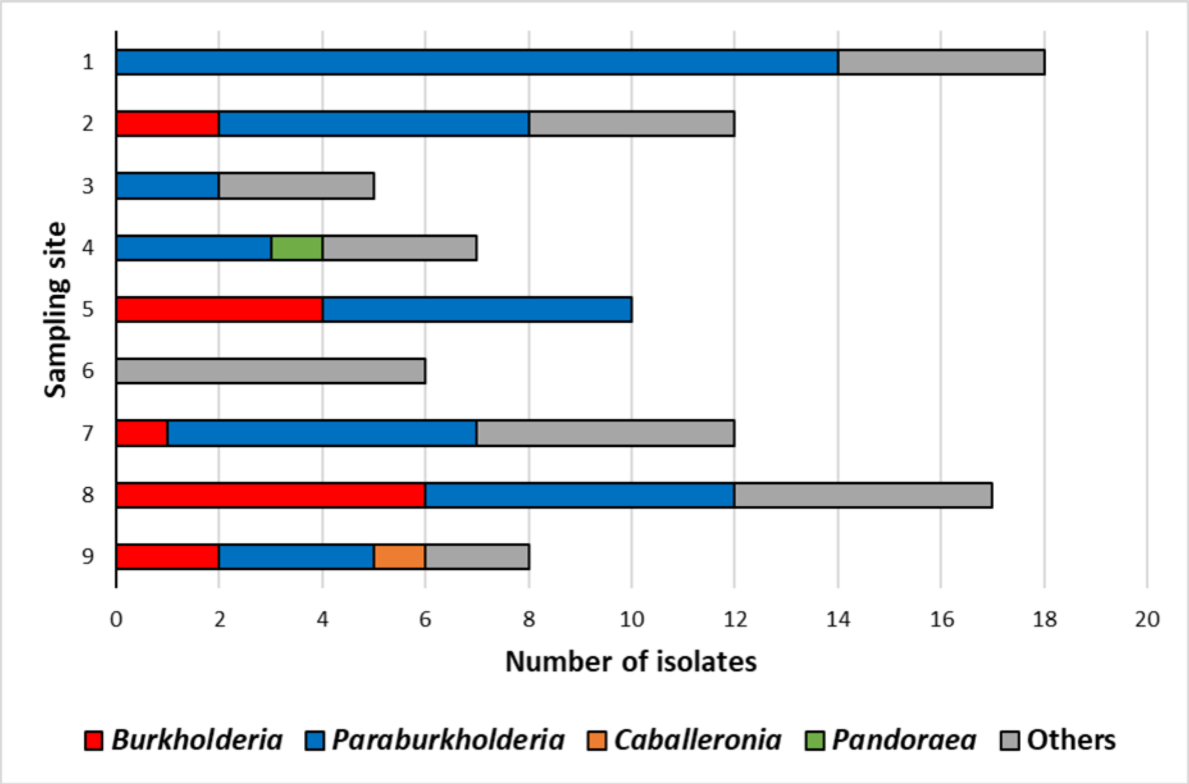

Supplementary Figure S3. Diversity of rainforest rhizosphere *Burkholderiaceae* isolates from nine different sampling sites (See Supplementary Figure S1) in the DGFC. The figure shows data on the diversity of the final rainforest isolate collection of 95 isolates. Taxonomic identification to genus level was based on bacterial 16S rRNA genes. The term ‘others’ represents the collective non-*Burkholderiaceae* genera recovered.

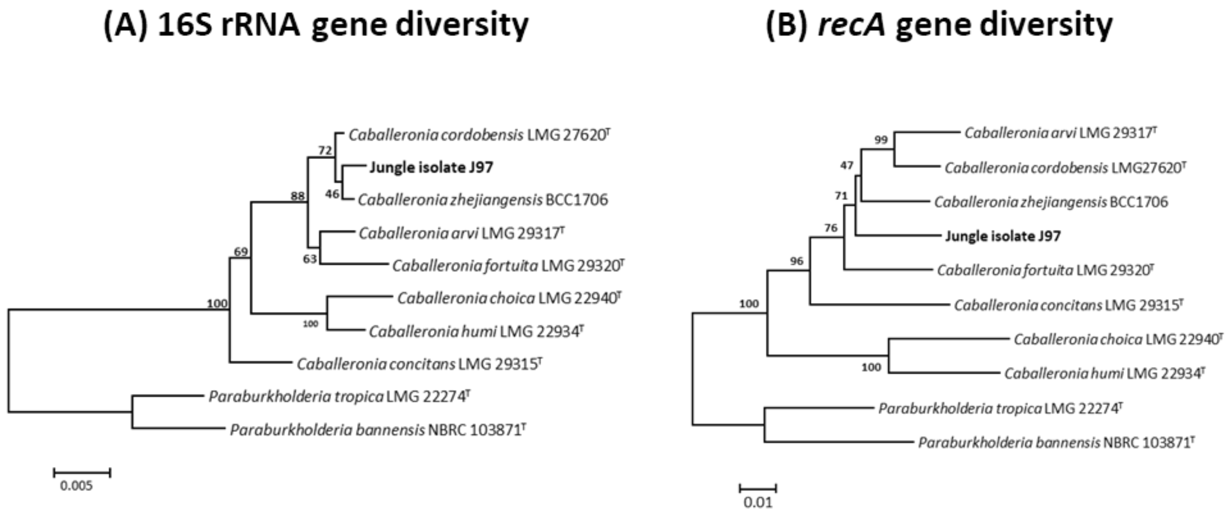

Supplementary Figure S4. Phylogenetic placement of the rainforest rhizosphere *Caballeronia* isolate J97 using the 16S rRNA and *recA* gene sequences. Clustering of the 16S rRNA gene (A) and *recA* gene (B) sequences is shown in relation to each other and the gene sequences from representative taxonomic type species. Trees were constructed using Minimum Evolution method and evolutionary distances computed using LogDet method. All positions containing gaps and missing data were eliminated and there were a total of 1525 and 1071 positions in the final datasets, respectively. *Paraburkholderia tropica* LMG 22274 and *Paraburkholderia bannensis* NBRC 103871 16S rRNA and *recA* gene sequences were used as outgroups. The percentage of replicate trees in which the associated taxa clustered together in the bootstrap test (1000 replicates) are shown next to the branches. Rainforest isolate identified in bold.

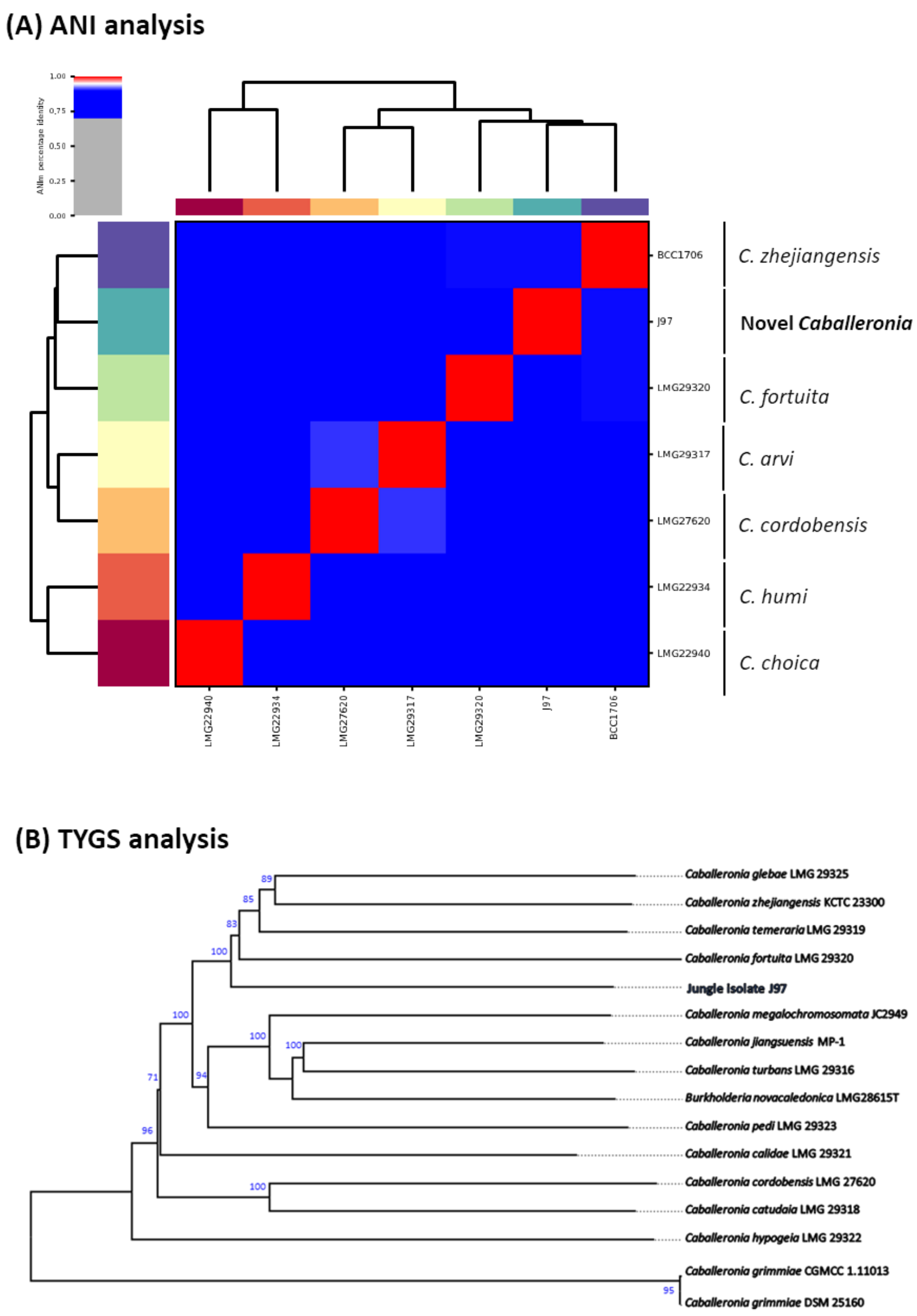

Supplementary Figure S5. Genomic taxonomy of rainforest rhizosphere *Caballeronia* isolate J97. Panel **(A)** shows the genome sequence taxonomic placement of rainforest rhizosphere *Caballeronia* isolate J97 inferred by average nucleotide identity (ANI). A heatmap generated by the PyANI script is shown and indicates the degree of nucleotide-level similarity between *Caballeronia* isolate J97 and *Caballeronia* reference strains. Colour-coding indicates the degree of nucleotide similarity, with red areas indicating >95% ANI, and darker shades of red indicating greater similarity. Blue indicates 70-95% ANI. **(B)** Phylogenomic analysis of *Caballeronia* isolate J97. The closest related *Caballeronia* type strain genomes were used for analysis as described by TYGS. The tree was inferred with FastME 2.1.6.1 using Genome BLAST Distance Phylogeny (GBDP) distances calculated from genome sequences. The branch lengths are scaled in terms of GBDP distance formula d5. The numbers above branches are GBDP pseudo-bootstrap support values >60% from 100 replications. The tree was rooted at the midpoint.

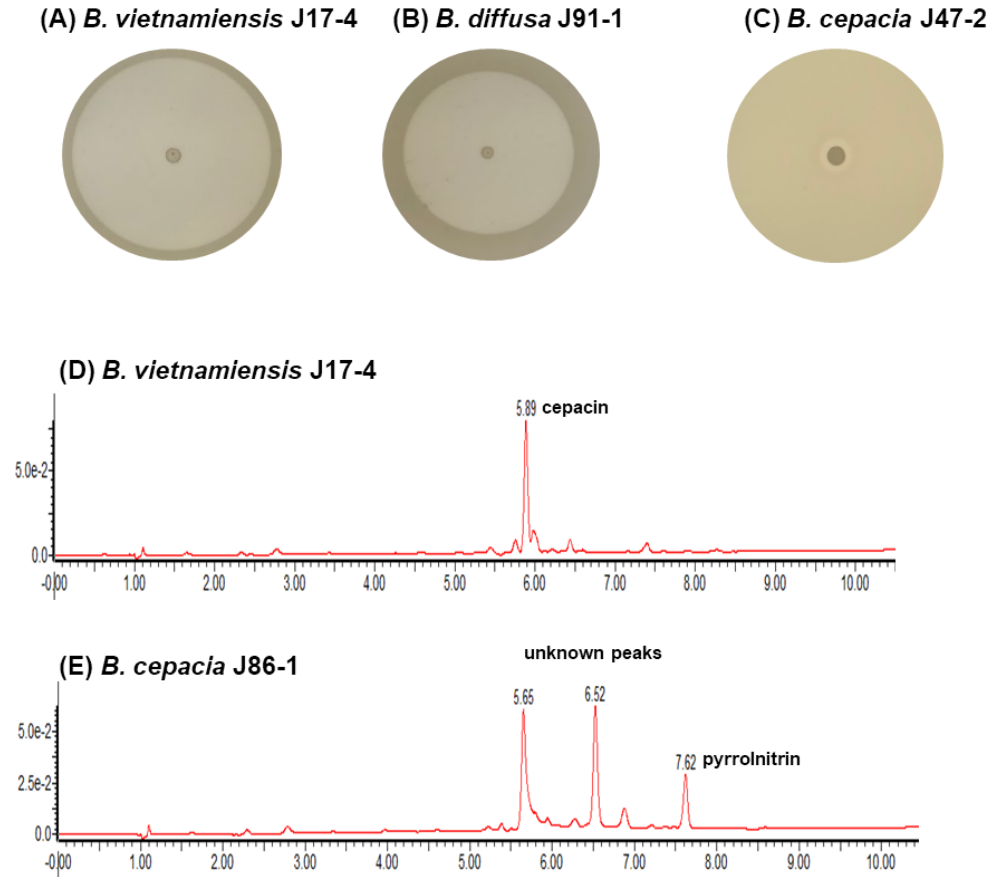

Supplementary Figure S6. Antimicrobial bioactivity and metabolite characterization of selected rainforest *Burkholderia.* An microbial overlay antagonism assay of the rainforest *Burkholderia* isolates was performed after 5 days of growth on BSM-G (pH 5) at 22°C as described. **(A-C)** Antimicrobial activity of each strain towards a methicillin resistant strain of *Staphylococcus aureus* is shown. **(D-E)** Examples of the metabolites produced and detected by HPLC from rainforest *Burkholderia* after growth on BSM-G (pH7), with the known metabolites (cepacin and pyrrolnitrin) and unknown metabolites indicated.

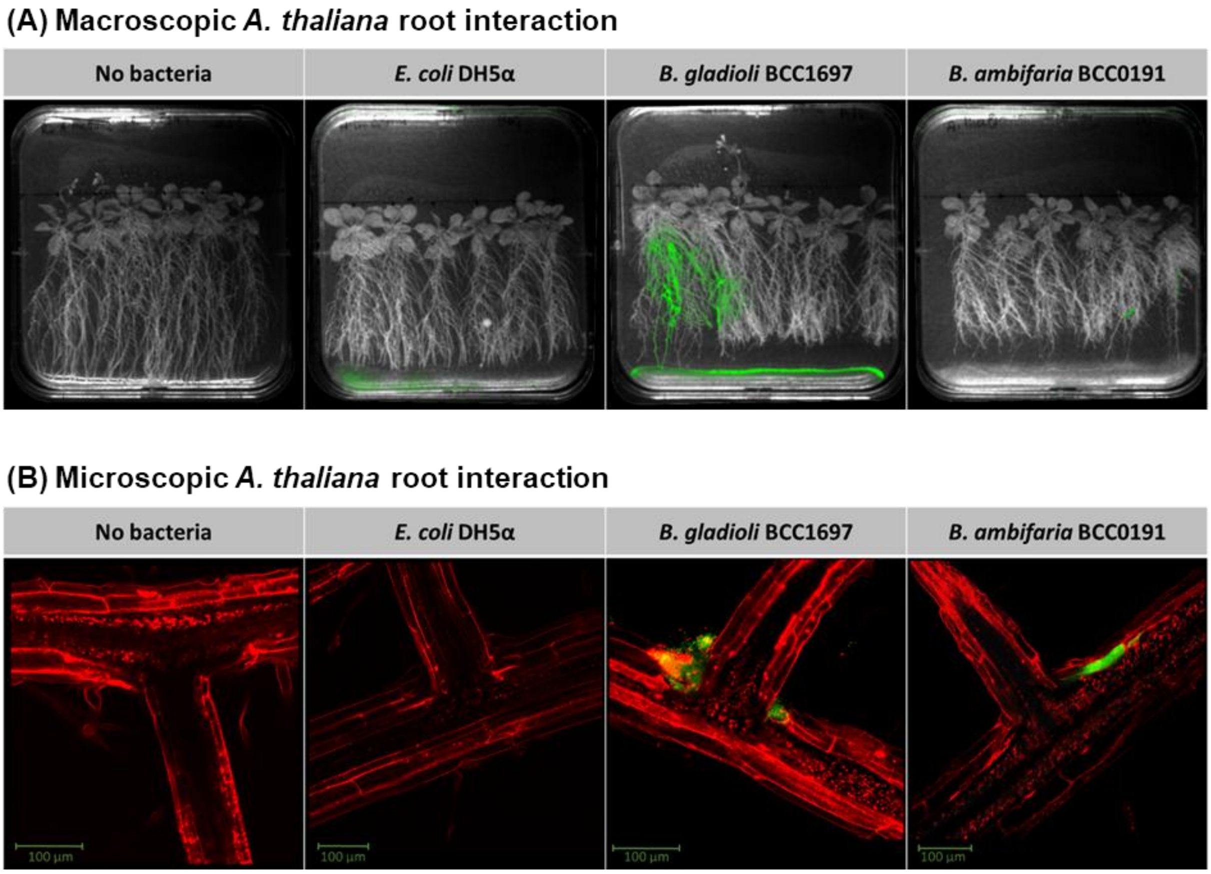

Supplementary Figure S7. *Burkholderia* macroscopic and microscopic root colonisation interactions in the *A. thaliana* rhizosphere interaction model. (A) For macroscopic plant interaction analysis, *A. thaliana* seedlings were grown vertically in the root colonisation model (2 weeks) and the fluorescently labelled *Burkholderia* suspension in soft agar was applied to the lower edge of the plate without touching the roots. After another 7 days vertical incubation (post-inoculation), the plates were imaged using a Biospace Labs PhotonIMAGER Optima at excitation wavelength 488 nm and emission wavelength 522 nm. The images are representative of eight different plates examined in four independent experiments. **(B)** To understand the cellular plant interaction phenotype of the *Burkholderia*, confocal microscopy was performed as follows. 7 days post the bacterial soft agar inoculation (Panel A), individual seedlings were removed from the plates, washed with PBS, stained with propidium iodide, immediately placed on a microscope slide with a cover slip and imaged using a Zeiss LSM 710 inverted confocal microscope with the following settings: excitation with 488 nm laser (4.5%), emission channel 1 (499-529 nm), emission channel 2 (595-719 nm), 44 µm confocal pinhole and Plan-Apochromat 20x/0.8 M27 objective. The images are representative of ten different fields examined for each bacteria-plant interaction.

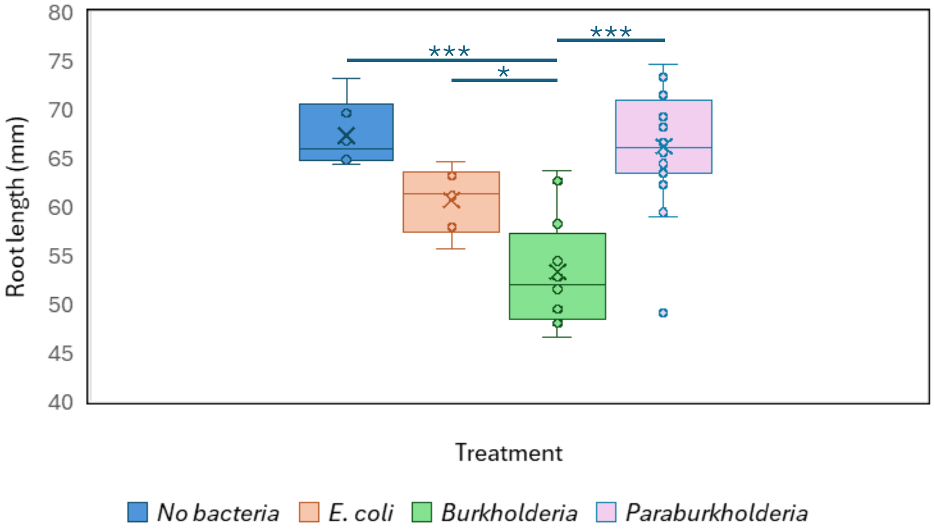

Supplementary Figure S8. The presence of *Burkholderia* significantly delays *A. thaliana* root growth within the rhizosphere interaction model. The *A. thaliana* root length after 14 days of growth was measured and statistically evaluated (ANOVA and Tukey's HSD testing) as described in the Methods. Plants within growth plates containing no bacteria or *Paraburkholderia* showed equivalent growth. Plants within the *Burkholderia*-inoculated growth plates showed a significant delay in root growth compared to the no bacteria control and *Paraburkholderia* groups (***; $p < 0.001$) and the *E. coli*-inoculated groups (*; $p < 0.05$).

Supplementary Table S1. Selected examples of pairwise ANI and *in silico* DDH values between rainforest isolates and their closest representative type strain.

Rainforest isolate	Pairwise ANI (%)[1]	Pairwise DDH (%)[2]
	Burkholderia diffusa CCUG 54558^T (GCA_008802145)	
J48	95.6	63.5
J91-1	95.6	63.7
J91-2	95.6	63.7
	Paraburkholderia bannensis NBRC 103871^T (GCA_008802145)	
J11-1	95.8	65.5
J15-1	95.8	64.9
J15-2	95.8	65.5
J35	96.1	67.4
J72	95.8	65.2
J74	95.8	65.0
J75-1	95.8	65.5
J75-2	95.8	65.1
	Paraburkholderia guartelaensis CNPSo3008^T (GCA_004353905)	
J10-2	96.3	68.4

[1] Average nucleotide identity (ANI) values <95% indicates different species; [2] *in silico* DNA-DNA hybridisation (DDH) values <70% indicates different species.

Supplementary Table S2. HPLC analysis of *Burkholderia* rainforest isolates demonstrating the presence of known and unidentified specialised metabolites

**HPLC detection of specialised metabolites**			
**Rainforest isolate**	**Cepacin**	**Pyrrolnitrin**	**Unidentified peaks (RT)[1, 2]**
B. vietnamiensis J17-1	+	-	1 peak (6.58)
B. vietnamiensis J17-4	+	-	1 peak (6.58)
B. cepacia J47-2	-	+	7 peaks (3.18, 5.67, 6.28, 6.54, 6.89, 7.23, 7.94)
B. cepacia J47-3	-	+	7 peaks (3.36, 3.98, 5.66, 6.27, 6.54, 6.88, 7.22)
B. cepacia J49	-	+	7 peaks (3.36, 5.67, 6.28, 6.54, 6.88, 7.23, 7.93)
B. cepacia J70	-	+	6 peaks (3.35, 5.67, 6.28, 6.55, 6.89, 7.24)
B. cepacia J80-2	-	+	4 peaks (5.67, 6.54, 6.88, 7.23)
B. cepacia J86-1	-	+	2 peaks (5.65, 6.52)
B. cepacia J86-2	-	+	4 peaks (5.67, 6.54, 6.88, 7.22)
B. diffusa J48	-	-	-
B. diffusa J91-1	+	-	2 peaks (6.95, 7.40)
B. diffusa J91-2	+	-	2 peaks (6.95, 7.40)

[1] RT = retention time measured by PDA at max plot (absorbance = 210-400 nm)

[2] Only peaks significantly above the baseline were reported in the analysis

Supplementary Table S3 Plant growth promotion properties of selected *Paraburkholderia* rainforest isolates

Plant promotion properties				
**Rainforest isolate**	**IAA production[1] (µg/ml) (n = 3)**	**Alfalfa root length[2] (mm) (n = 96)**	**Phosphate solubilization[3] (mm) (n = 4)**	**Growth on N-free medium[4]**
Paraburkholderia bannensis J75-1 (BCC1915)	1.61 ± 0.35	31.32 ± 10.88	9.25 ± 0.50	+
Paraburkholderia bannensis J75-2	ND	ND	8.63 ± 0.48	+
Paraburkholderia tropica J19-1 (BCC1950)	4.10 ± 1.35	32.73 ± 12.06	10.50 ± 0.58	+
P. tropica LMG 22274^T	3.35 ± 1.32	35.96 ± 14.07	11.25 ± 0.50	+
P. phytofirmans PsJN (LMG 22146^T)	2.48 ± 0.73	30.43 ± 12.21	8.38 ± 0.48	+
Untreated control	-	29.88 ± 11.04	-	-

Values shown are mean ± SD; ND = not determined; - = not required for this assay; Untreated control = alfalfa seeds without any bacterial inoculum.

[1] IAA (indole-3-acetic acid) production measured using a modified Salkowski's reagent spectrophotometric method.

[2] Alfalfa root length measured after 10 days growth.

[3] Phosphate solubilisation was measured as the diameter (mm) of the clear zone surrounding the bacterial colony.

[4] Growth on N-free medium was assessed on BSMG without a N source.
